# Supplementary material for: A contingent valuation experiment about future particle accelerators at CERN
Source: PLoS One. 2020 Mar 11;15(3):e0229885. doi: 10.1371/journal.pone.0229885 (PMC7065825; doi:10.1371/journal.pone.0229885)
Supplement: S1 File — (PDF) [file pone.0229885.s001.pdf]

SUPPORTING INFORMATION
A contingent valuation experiment about future particle accelerators at CERN

S1 File. Pilot experiments and pre-tests

The final version of the CV questionnaire used in this study is the result of developing and designing activities implemented between June 2014 and January 2018.

The first pilot experiment [1] was conducted in the period June 2014-March 2015 and involved 1,022 students in five European universities and four countries: University of Milan (Italy), University of A Coruña (Spain), University Paris 7- Denis Diderot and Sciences Po University (France), and University of Exeter (UK). 40% of interviewed students were form Italy, and 20% from Spain, France and the UK each. They were enrolled in more than 30 different university degrees covering both social sciences and humanities (65.1%) and science, technology, engineering and mathematics curricula (34.9%). 57% were female. In that pilot test the main goal was the identification of the individual socio-economic and cultural characteristics affecting individual preferences for particle physics research carried out at the Large Hadron Collider (LHC) at CERN (our sample was not representative of the students' population in the countries). The questionnaire was structured in three sections bearing, respectively, on: (i) attitudes and broad awareness of research infrastructures; (ii) description of the LHC with photos followed by questions on WTP; (iii) sociodemographic characteristics of respondents. Students' willingness to financially contribute to that research was investigated in different ways. The first question was qualitative and asked their willingness to pay without mentioning any bid: "Would you be willing to provide an economic contribution to fund the research activity of the LHC?" with answer options "yes" ticked by 19% of respondents, "no" (by 33%) and "I do not know" (by 48%). After, two follow-up questions were submitted to respondents. The first one asked the willingness to pay a lump-sum payment of EUR 30 "By 2015, would you be willing to offer an economic contribution equal to EUR 30, renouncing other personal expenses?" with the amount of EUR 30 coming from a literature review covering one hundred WTP studies on environmental, health and cultural goods either using stated or revealed preferences approaches. The bid offered reduced the percentage of "yes" to 15% and increased the percentage of "no" to 49%. The second follow up question asked to choose an annual payment as follows: "If you are asked to give an economic contribution to the LHC by means of an annual tax over a period of 30 years, would you be willing to pay an annual amount equal to EUR 0 (not paying at all), EUR 0.5, EUR 1 or EUR 2?" The share of students who chose EUR 0 was 27%; in contrast, the remaining 73% ticked a positive amount with EUR 0.5 chose by 8% of them, EUR 1 by 22%, and EUR 2 by 43%. Whatever the question, respondents were also asked to explain their choice by filling in an open-ended question while data were further analyzed by multinomial or ordered logit econometric models according to the question under analysis. Such a first exploratory experiment delivered two main findings. The first was that respondents are likely to react differently to various payment proposals (lump-sum versus annual payments) [2, 3, 4]. Although many factors may be responsible, in our opinion the preference for smaller annual payments with respect to a larger lump-sum payment was just the result of a mental accounting [4]. Budget constraints are likely to constrain in either case; however, respondents feel less bound when are asked smaller ongoing payments rather than larger lump-sum disbursement. An additional reason may be that, when dealing with donations to charities or organisations, included science-related ones, people are used to donating a fixed amount each year instead of a large one-time payment. The second was that the WTP for investment

in basic science was driven by the (family) income, that is that greater the income, the more likely was to obtain a “yes” response, by personal attitudes such as being interested in scientific research and previous awareness of what CERN and the LHC are. This evidence holds regardless the type of course students were enrolled in.

The second pilot experiment [5] involved 230 students in economics at University of Milan and was conducted in two steps: 120 students were surveyed in June 2016 and 110 students were interviewed after one year in June 2017. Differently from the first pilot experiment, the main goal was to test the amount of an array of bids and get an idea of the resulting WTP, therefore a referendum SBDC-CV-like survey was administered. The exact wording of the WTP question was: “Would you be willing to pay EUR _ every year to fund the research activity at LHC turning down other personal expenses?” with answers “yes” or “no”. Six anonymized versions of the questionnaire were randomly distributed with one of the following pre-printed bids: EUR 1, EUR 2, EUR 5, EUR 10, EUR 15, and EUR 30. Both closed- and open- ended questions were asked to respondents to explain why they chose to fund or not to fund the program. Just before answering the WTP questions, students were provided with a shortened description of the LHC taken from Wikipedia entry “Large Hadron Collider” including five photos. The questionnaire also informed that investments projects at CERN are funded by CERN member States by means of taxation and therefore they were made aware that citizens indirectly supports basic science projects and public contributions enable CERN to continue its research activity. However, the questionnaire did not disclose the actual per-capita amount of the Italian government contribution to the CERN budget to avoid anchoring. In the whole sample, 57% of students were male, 49% declared having heard about LHC before the experiment and 74.8% had heard about the Higgs boson; in contrast, only 5.7% visited CERN. The distribution of respondents according to their monthly family income was: 42.3% belonged to a family with a monthly income between EUR 1,000 and 3,000; 37.7% between EUR 3,000 and 5,000; 16.3% more than EUR 5,000 and 3.7% respondents belong to a family earning less than EUR 1,000. The first bid of EUR 1 received 100% of “yes” responses; while the last bid of EUR 30 was rejected only by 27% of respondents (73% of “yes”). Data analysis resorted to both parametric models such as the logit model and the non-parametric pooling adjacent violator algorithm by Ayer et al. [6]. The truncated mean WTP, obtained through the logit model and integrating the resulting logistic curve from 0 to the maximum bid of EUR 30, was EUR 28 per person per year; while the non-parametric estimation yielded a lower mean WTP of EUR 23 per person per year. The experiment revealed that the WTP for basic research is negatively influenced by the bid asked (the higher the bid, the lower the probability of answering “yes”) and positively associated with income: students that were members of families with a higher income were willing to pay more. This second experiment also confirmed findings from the first experiment. Respondents who stated to be interested in scientific research showed a higher probability to answer “yes” with respect to colleagues not interested in research; having heard about CERN or its discoveries such as the Higgs boson were positively associated with the WTP.

References

1. Florio M, Giffoni F, Catalano G. Should governments fund basic science? Evidence from a willingness-to-pay experiment in five universities. Journal of Economic Policy Reform. 2018; 1-18 doi.org/10.1080/17487870.2018.1547638

2. Stevens TH, DeCoteau NE, Willis CE. Sensitivity of contingent valuation to alternative payment schedules. Land Economics. 1997; 73:140-8. DOI: 10.2307/314 https://www.jstor.org/stable/3147083
3. Echeverría J, Hanrahan M, Solórzano R. Valuation of non-priced amenities provided by the biological resources within the Monteverde Cloud Forest Preserve, Costa Rica. Ecological Economics. 1995; 13(1):43-52. https://doi.org/10.1016/0921-8009(95)00054-D
4. Egan KJ, Corrigan JR, Dwyer DF. Three reasons to use annual payments in contingent valuation surveys: Convergent validity, discount rates, and mental accounting. Journal of Environmental Economics and Management. 2015; 72:123-36. https://doi.org/10.1016/j.jeem.2015.05.002
5. Florio M, Giffoni F. Willingness-to-pay for science as a public good: a contingent valuation experiment. Working Paper 17/2017, Department of of Economics, Management and Quantitative Methods, University of Milan. 2017. Available from: http://wp.demm.unimi.it/files/wp/2017/DEMM-2017_17.pdf
6. Ayer M, Brunk HD, Ewing GM, Reid WT, Silverman E. An empirical distribution function for sampling with incomplete information. The Annals of Mathematical Statistics. 1955; 26: 641-647 https://www.jstor.org/stable/2236377
