# Supplementary material for: A contingent valuation experiment about future particle accelerators at CERN
Source: PLoS One. 2020 Mar 11;15(3):e0229885. doi: 10.1371/journal.pone.0229885 (PMC7065825; doi:10.1371/journal.pone.0229885)
Supplement: S2 File — (PDF) [file pone.0229885.s002.pdf]

SUPPORTING INFORMATION
A contingent valuation experiment about future particle accelerators at CERN

S2 File. The questionnaire (in French and in English)

ENQUÊTE SUR LE SOUTIEN DE LA POPULATION À LA RECHERCHE SCIENTIFIQUE AU CERN

SECTION A : VOS CENTRES D'INTERETS

A.1 Dans quelle mesure êtes-vous intéressé(e) par les sujets suivants ? Répondez aux questions en cochant la case correspondant à votre choix.

	Pas du tout	*Très peu*	*Un peu*	*Assez*	*Beaucoup*
A.1.1 Sports	☐	☐	☐	☐	☐
A.1.2 Politique et Société	☐	☐	☐	☐	☐
A.1.3 Biologie	☐	☐	☐	☐	☐
A.1.4 Physique	☐	☐	☐	☐	☐
A.1.5 Astronomie	☐	☐	☐	☐	☐
A.1.6 Géologie	☐	☐	☐	☐	☐
A.1.7 Médicine	☐	☐	☐	☐	☐
A.1.8 Environnement	☐	☐	☐	☐	☐
A.1.9 Arts et culture	☐	☐	☐	☐	☐

A.2 A quelle fréquence consultez-vous les médias ci-dessous pour suivre l'actualité des sujets qui vous intéressent ?

	Jamais	*Occasionnellement*	*Souvent*
A.2.1 Télévision	☐	☐	☐
A.2.2 Radio	☐	☐	☐
A.2.3 Presse écrite	☐	☐	☐
A.2.4 *Livres*	☐	☐	☐
A.2.5 Internet et médias sociaux	☐	☐	☐

A.3 Pour chaque affirmation, cochez la case correspondant à votre choix

**La recherche scientifique est importante pour :**	*Pas du tout d'accord*	*Pas d'accord*	*Sans opinion*	*D'accord*	*Tout à fait d'accord*
A.3.1 améliorer la santé et la qualité de vie	☐	☐	☐	☐	☐

**A.3.2** assurer le futur des générations à venir	☐	☐	☐	☐	☐
**A.3.3** satisfaire la curiosité humaine sur la nature et les origines de l'Univers	☐	☐	☐	☐	☐
**A.3.4** soutenir la croissance économique et l'emploi	☐	☐	☐	☐	☐
**A.3.5** encourager la création de nouveaux produits et services	☐	☐	☐	☐	☐

**A.4** Parmi les organisations internationales citées ci-contre, quelles sont celles que vous connaissez ?<br><br>*(Si vous cochez la case CERN, passez directement à la question A.5 autrement passez à la section B)*	☐ OMS (Organisation mondiale de la santé)<br>☐ NASA (Administration nationale de l'aéronautique et de l'espace)<br>☐ CERN (l'Organisation européenne pour la recherche nucléaire)<br>☐ ASE (Agence spatiale européenne)<br>☐ FMI (Fond Monétaire International)<br>☐ CEA (Commissariat à l'énergie atomique et aux énergies alternatives.)<br>☐ CNRS (Centre national de la recherche scientifique)<br>☐ FAO (Organisation des Nations unies pour l'alimentation et l'agriculture)<br>☐ ESRF (Installation européenne de rayonnement synchrotron)<br>☐ UNESCO (Organisation des Nations Unies pour l'éducation, la science et la culture)

**A.5** Vous connaissez le CERN parce que…<br><br>*(choix multiple possible)*	☐ Vous avez entendu parler du CERN par le biais d'une personne (ex. collègues, amis, membres de la famille, employés du CERN, …)<br>☐ Vous avez participé à des événements culturels organisés par le CERN ou en rapport avec le CERN<br>☐ Vous avez lu des informations ou des livres mentionnant le nom du CERN<br>☐ Vous avez visionné des films et des documentaires sur le CERN<br>☐ Vous avez visité le CERN<br>☐ Autres (précisez) :<br>☐ ______________________________

**A.6** Quand vous pensez au CERN, qu'est-ce que cela vous évoque en premier lieu ?

**A.7** Quel est votre avis sur le CERN ?<br><br>Cochez la case correspondant à votre choix	☐ Positif<br>☐ Plutôt positif<br>☐ Neutre<br>☐ Plutôt négatif

	☐ Négatif

SECTION B : VOTRE CONNAISSANCE DU CERN

Cliquez le lien pour lire ce document de 2 pages sur le CERN : lien

Cliquez ici pour visionner cette vidéo d'environ 2 minutes sur le CERN : https://cds.cern.ch/record/2292503

**B.1** Quels sont les aspects du CERN que vous appréciez ?	
**B.2** Quels sont les aspects du CERN que vous n'appréciez pas ?	
**B.3** Quelle est votre perception sur le CERN ?<br><br>Cochez la case correspondant à votre choix	☐ Positif<br>☐ Plutôt positif<br>☐ Neutre<br>☐ Plutôt négatif<br>☐ Négatif

B.4

Pour chacune des affirmations suivantes, cochez la case correspondant à votre choix

	Pas du tout d'accord	*Pas d'accord*	*Sans opinion*	*D'accord*	*Tout à fait d'accord*
B.4.1 Le CERN développe de nouvelles technologies pour le diagnostic et le traitement de maladies	☐	☐	☐	☐	☐
B.4.2 Les découvertes du CERN nous permettent d'enrichir notre connaissance sur les origines et l'évolution de l'Univers	☐	☐	☐	☐	☐
B.4.3 Le CERN est une organisation d'aide humanitaire	☐	☐	☐	☐	☐
B.4.4 Les découvertes du CERN peuvent déboucher sur la création de produits qui	☐	☐	☐	☐	☐

pourraient améliorer la qualité de vie					

**B.5** Pour chacune des affirmations suivantes, cochez la case correspondant à votre choix					
La recherche au CERN …	*Pas du tout d'accord*	*Pas d'accord*	*Sans opinion*	*D'accord*	*Tout à fait d'accord*
B.5.1…est importante pour les scientifiques uniquement	☐	☐	☐	☐	☐
B.5.2 … est importante pour tout le monde	☐	☐	☐	☐	☐
B.5.3 … est importante pour les habitants de la région environnante uniquement	☐	☐	☐	☐	☐
B.5.4 … est dangereuse pour l'environnement	☐	☐	☐	☐	☐

**B.6** Pour chacune des affirmations suivantes, cochez la case correspondant à votre choix					
	Pas du tout d'accord	*Pas d'accord*	*Sans opinion*	*D'accord*	*Tout à fait d'accord*
B.6.1 Je suis fier(e) que le CERN soit implanté aussi sur le sol français	☐	☐	☐	☐	☐
B.6.2 La recherche au CERN agit positivement sur ma vie de tous les jours	☐	☐	☐	☐	☐
B.6.3 L'activité de recherche au CERN devrait s'intensifier au cours des prochaines décennies	☐	☐	☐	☐	☐
B.6.4 Le programme d'éducation développé au CERN pour les étudiants et les jeunes professionnels crée de la valeur à notre société	☐	☐	☐	☐	☐
B.6.5 Les activités de recherche internationale du CERN contribuent à la paix dans le monde	☐	☐	☐	☐	☐

SECTION C: VOTRE SOUTIEN AU CERN

La recherche avec les accélérateurs de particules, dont le grand collisionneur de hadrons (*Large Hadron Collider – LHC*) du CERN, a permis d'établir une représentation théorique de l'Univers. Cependant, les recherches mettent en évidence des phénomènes qui ne peuvent pas être expliqués par cette théorie.

Les Etats membres du CERN, dont la France, financent cette recherche. Voici deux scénarios possibles quant à l'avenir de cette recherche.

Scenario A	Scenario B
Les Etats membres du CERN décident d'investir dans un nouvel accélérateur de particules dans les dix ans à venir. Il permettra de faire des découvertes sur des phénomènes qui ne peuvent pas être expliqués aujourd'hui. Ce nouvel accélérateur sera exploité pendant au moins vingt-cinq ans.	Les Etats membres du CERN décident de ne pas investir dans un nouvel accélérateur de particules. L'activité de recherche avec l'accélérateur existant, le LHC, diminuera progressivement au cours des vingt prochaines années. La possibilité de trouver des réponses sur des phénomènes non-expliqués restera limitée.

VOTRE CHOIX

C.1 Accepteriez-vous de payer **la somme de ____ euros par an en tant que contribuable** pour la construction d'un nouvel accélérateur de particules au CERN tel que décrit dans le scénario A?

OUI (Passez à la question C.5) NON (Passez à la question C.2)

C.2 Accepteriez-vous de payer **une somme de ____ euros par an en tant que contribuable** par an pour soutenir cet investissement ?

OUI (Passez à la question C.3.1) NON (Passez à la question C.3.2)

C.3.1 Quelle serait votre **participation annuelle maximale en tant que contribuable** pour soutenir cet investissement ?

Je participerai à hauteur de ____ euros
(Passez à la question C.6)

C.3.2 Quelle serait votre **participation annuelle maximale en tant que contribuable** pour soutenir cet investissement ?

Je ne participerai pas financièrement
(Passez à la question C.4)

Je participerai à hauteur de ____ euros
(Passez à la question C.6)

C.4 (Répondez à cette question uniquement si vous avez coché la case précédente "Je ne participerai pas financièrement".
Quelle sont les principales raisons pour lesquelles vous ne participeriez pas financièrement ? Choisissez un maximum de deux options.

☐ Je préfère dépenser mon argent pour d'autres choses
☐ Je n'ai actuellement pas les moyens de participer financièrement
☐ Je ne comprends pas la finalité des recherches scientifiques du CERN Par conséquent je ne souhaite pas participer financièrement
☐ Je suis contre les programmes financés par les gouvernements
☐ Je suis contre les organisations internationales
☐ Je pense que mon argent devrait être utilisé pour financer d'autres activités de recherche
☐ Seules les personnes profitant directement des activités de recherche du CERN devraient participer financièrement
☐ Autres raisons (précisez) :
____
____

ALLEZ A LA SECTION D

C.5 Accepteriez-vous de payer **une somme de ____ euros par an en tant que contribuable** par an pour soutenir cet investissement ?

☐ OUI (Passez à la question C.5.1)   ☐ NON (Passez à la question C.5.1)

C.5.1 Quelle serait votre **participation annuelle maximale ?**

Je participerai à hauteur de ____ euros

C.6 Pour quelle(s) raison(s) payeriez-vous ce montant ? Choisissez un maximum de deux options.

☐ Je considère que les activités du CERN justifient au moins ce montant
☐ À travers cette recherche les qualifications de toutes les personnes impliquées dans les projets s'accroissent
☐ La recherche permet aux entreprises de développer de nouveaux produits et services
☐ On devrait toujours soutenir ce type de recherche, même si les résultats n'aboutissent pas forcement à une application concrète
☐ La France bénéficie des activités du CERN
☐ L'Europe bénéficie des activités du CERN
☐ Le monde entier bénéficie des activités du CERN
☐ Autres raisons (précisez) :
____
____

SECTION D: VOTRE PROFIL	
**D.1** Vous êtes	☐ un homme<br>☐ une femme
**D.2** Votre âge	____________________ ans
**D.3** Votre plus haute qualification	☐ Niveau 6 ou 5 bis : collège ou passage en filière CAP / BEP<br>☐ Niveau 5 : BEP / CAP<br>☐ Niveau 4 : Brevet Professionnel / Bac Pro ou Bac Technologique / écoles des formations sanitaires ou sociales<br>☐ Niveau 3 : DUT / BTS<br>☐ Niveau 2 : Licence / Master universitaire<br>☐ Niveau 1 : Ingénieur / Master de Grande Ecole / Doctorat
**D.4** Votre métier	☐ Cadre<br>☐ Employé<br>☐ Ouvrier<br>☐ Artisan<br>☐ Indépendant<br>☐ Sans activité<br>☐ Retraité(e)<br>☐ Etudiant(e)<br>☐ Scientifique/Chercheur<br>☐ Ingénieur<br>☐ Enseignant(e)<br>☐ Autre (précisez):<br>____________________
**D.5** Votre région	☐ Auvergne - Rhône-Alpes<br>☐ Bourgogne - Franche-Comté<br>☐ Bretagne<br>☐ Corse<br>☐ Centre - Val de Loire (Centre)<br>☐ Grand Est (Alsace, Champagne Ardenne, Lorraine)<br>☐ Hauts de France (Nord Pas-de-Calais - Picardie)<br>☐ Île de France<br>☐ Nouvelle Aquitaine (Aquitaine, Poitou-Charentes, Limousin)<br>☐ Normandie (Basse Normandie and Haute Normandie)<br>☐ Occitanie (Midi-Pyrénées, Languedoc- Roussillon)<br>☐ Pays de la Loire<br>☐ Provence-Alpes-Côte d'Azur

**D.6** Votre zone d'habitation	☐ Rurale<br>☐ Urbaine - périphérique
**D.7** Le montant de votre **salaire mensuel net**	☐ en dessous de € 1,000<br>☐ € 1,001 - 1,200<br>☐ € 1,201 – 1,500<br>☐ € 1,501– 1,800<br>☐ € 1,801 – 2,000<br>☐ €2,001– 2,300<br>☐ € 2,301– 3,000<br>☐ €3,001– 4,000<br>☐ € 4,000 – 5,000<br>☐ € 5,001 – 6,000<br>☐ € 6,001 – 7,000<br>☐ € 7,001 – 8,000<br>☐ € 8,001 – 9,000<br>☐ 9,001 et plus
**D.8** Votre ménage	Nombre d'adultes :<br><br>Nombre d'enfants (< 18 ans) :

MERCI POUR VOTRE PARTICIPATION A L'ENQUÊTE

Vous pouvez ajouter des commentaires dans l'espace ci-dessous :

UNIVERSITÀ DEGLI STUDI DI MILANO

SURVEY ON SUPPORT OF THE POPULATION TO THE SCIENTIFIC RESEARCH AT CERN

SECTION A: YOUR INTERESTS

A.1 How much interested are you in the following topics? Answer the questions by ticking the box corresponding to your choice.

	Not at all	*Not much*	*Fairly*	*Much*	*Very much*
A.1.1 Sports	☐	☐	☐	☐	☐
A.1.2 Politics and Society	☐	☐	☐	☐	☐
A.1.3 Biology	☐	☐	☐	☐	☐
A.1.4 Physics	☐	☐	☐	☐	☐
A.1.5 Astronomy	☐	☐	☐	☐	☐
A.1.6 Geology	☐	☐	☐	☐	☐
A.1.7 Medicine	☐	☐	☐	☐	☐
A.1.8 Environment	☐	☐	☐	☐	☐
A.1.9 Arts and culture	☐	☐	☐	☐	☐

A.2 How often do you watch/read the media below to follow news on topics you are interest in?

	Never	*Occasionally*	*Often*
A.2.1 Television	☐	☐	☐
A.2.2 Radio	☐	☐	☐
A.2.3 Newspaper	☐	☐	☐
A.2.4 Books	☐	☐	☐
A.2.5 Internet and social media	☐	☐	☐

A.3 For each statement, tick the box corresponding to your choice.

**Scientific research is important to:**	*Strongly disagree*	*Disagree*	*Neutral*	*Agree*	*Strongly agree*
A.3.1 Improve health and quality of life	☐	☐	☐	☐	☐
A.3.2 Secure the future of next generations	☐	☐	☐	☐	☐
A.3.3 Satisfy human curiosity about the nature and origin of the Universe	☐	☐	☐	☐	☐

A.3.4 Support economic growth and employment	☐	☐	☐	☐	☐
A.3.5 Encourage the creation of new products and services	☐	☐	☐	☐	☐

**A.4 Which of the following international organizations are you aware of?**<br><br>*(If you tick the CERN, go directly to question A.5 otherwise go to section B)*	☐ WHO (World Health Organization)<br>☐ NASA (National Aeronautics and Space Administration)<br>☐ CERN (European Organization for Nuclear Research)<br>☐ ESA (European Space Agency)<br>☐ IMF (International Monetary Fund)<br>☐ CEA (French Alternative Energies and Atomic Energy Commission)<br>☐ CNRS (French National Centre for the Scientific Research)<br>☐ FAO (Food and Agriculture Organization of the United Nations)<br>☐ ESRF (European Synchrotron Radiation Facility)<br>☐ UNESCO (United Nations Educational, Scientific and Cultural Organization)

**A.5 You know CERN because ...**<br><br>*(more than one answer is possible)*	☐ You heard about CERN from a person (e.g. colleagues, friends, family members, CERN employees, ...)<br>☐ You participated at cultural events organized by CERN or in connection with CERN<br>☐ You read books and news mentioning CERN<br>☐ You watched films and/or documentaries related to CERN<br>☐ You visited CERN<br>☐ Other (specify): ______________________

**A.6 When you think of CERN, what does it come to your mind first?**

**A.7 What is your opinion about CERN?**<br><br>*Tick the box corresponding to your choice*	☐ Positive<br>☐ Somewhat positive<br>☐ Neutral<br>☐ Rather negative<br>☐ Negative

SECTION B: YOUR KNOWLEDGE OF CERN

Click the link to read the document on CERN:
Click here to watch the 2-minute video about CERN:

B.1 What aspects of CERN do you like?

B.2 What aspects of CERN do you dislike?

**B.3 What is your perception of CERN?**<br><br>*Tick the box corresponding to your choice*	☐ Positive<br>☐ Somewhat positive<br>☐ Neutral<br>☐ Rather negative<br>☐ Negative

B.4 For each of the following statements, tick the box corresponding to your choice

	Strongly disagree	*Disagree*	*Neutral*	*Agree*	*Strongly agree*
B.4.1 CERN develops new technologies for the diagnosis and treatment of diseases	☐	☐	☐	☐	☐
B.4.2 CERN's discoveries allow us to enrich our knowledge of the origins and evolution of the Universe	☐	☐	☐	☐	☐
B.4.3 CERN is a humanitarian aid organization	☐	☐	☐	☐	☐
B.4.4 CERN's discoveries can lead to the creation of products that could improve the quality of life	☐	☐	☐	☐	☐

B.5 For each of the following statements, tick the box corresponding to your choice

Research at CERN …	*Strongly disagree*	*Disagree*	*Neutral*	*Agree*	*Strongly agree*
B.5.1… is important for scientists only	☐	☐	☐	☐	☐
B.5.2 … is important for everyone	☐	☐	☐	☐	☐
B.5.3 … is important only for people living in the surrounding area	☐	☐	☐	☐	☐

UNIVERSITÀ DEGLI STUDI DI MILANO

B.5.4 … is dangerous for the environment	☐	☐	☐	☐	☐

**B.6 For each of the following statements, tick the box corresponding to your choice**					
	Strongly disagree	*Disagree*	*Neutral*	*Agree*	*Strongly agree*
B.6.1 I am proud that CERN is also established on French soil	☐	☐	☐	☐	☐
B.6.2 Research at CERN has a positive effect on my everyday life	☐	☐	☐	☐	☐
B.6.3 Research activity at CERN should be intensified over the coming decades	☐	☐	☐	☐	☐
B.6.4 CERN's education programs for students and young professionals create value for society	☐	☐	☐	☐	☐
B.6.5 CERN's research activities contribute to the peace in the world	☐	☐	☐	☐	☐

SECTION C: YOUR SUPPORT AT CERN

Particle accelerator research, including the Large Hadron Collider (LHC) at CERN, has established a theoretical representation of the Universe. However, the research highlights phenomena that cannot be explained by this theory.

CERN Member States, including France, are financing this research.

Here are two possible scenarios for the future of this research.

Scenario A

CERN Member States decide to invest in a new particle accelerator in the next decade. It will make discoveries on phenomena that cannot be explained today. This new accelerator will be operated for at least twenty-five years.

Scenario B

CERN Member States decide not to invest in a new particle accelerator. The research activity with the existing accelerator, the LHC, will gradually decrease over the next twenty years. The possibility of finding answers on unexplained phenomena will remain limited.

YOUR CHOICE

C.1 Would you be in favour to **pay the sum of EUR ____ per year as a taxpayer** for the construction of a new particle accelerator at CERN as described in Scenario A?

YES **(Go to question C.5)** NO **(Go to question C.2)**

C.2 Would you be in favour to **pay the sum of EUR ____ per year as a taxpayer** to support such an investment?

YES **(Go to question C.3.1)** NO **(Go to question C.3.2)**

C.3.1 What is the **maximum annual amount you would pay as a taxpayer** for supporting this investment?

I would participate with EUR ____
(Go to question C.6)

C.3.2 What is the **maximum annual amount you would pay as a taxpayer** for supporting the investment ?

I would not participate financially at all
(Go to question C.4)

I would participate with EUR____
(Go to question C.6)

C.4 Answer this question only if you answered the previous question: "I would not participate financially at all".
What are the main reasons you would pay zero? Please, choose at most two options.

- ☐ I prefer to spend my money for other things
- ☐ Currently, I cannot afford this expense
- ☐ I do not understand the purpose of CERN's scientific research. Therefore, I do not want to contribute
- ☐ I am against government-funded programs
- ☐ I am against international organizations
- ☐ I think my money should be used to fund other research activities
- ☐ Only people who directly benefit from CERN research activities should pay for it
- ☐ Other reasons (specify):

____

GO TO THE SECTION D

C.5 Would you be in favour to **pay the sum of EUR ____ per year as a taxpayer** to support this investment?

YES **(Go to the question C.5.1)** NO **(Go to the question C.5.1)**

C.5.1 What is the **maximum annual amount you would pay as a taxpayer** for supporting this investment?

I would participate with EUR ____

C.6 For what reason (s), would you pay this amount? Please, Choose at least two options.

- ☐ I think that CERN's activities justify at least this amount
- ☐ Through this research the competences/skills of all people involved in the projects will grow
- ☐ Research enables companies to develop new products and services

- ☐ This type of research should be always supported, even if the results do not necessarily lead to any concrete application
- ☐ France benefits from CERN activities
- ☐ Europe benefits from CERN activities
- ☐ The whole world benefits from CERN activities
- ☐ Other reasons (specify) :

____

SECTION D: YOUR PROFILE

**D.1 You are**	☐ Male<br>☐ Female
**D.2 Your age**	____________ years
**D.3 Your highest qualification**	☐ Niveau 6 ou 5 bis : collège ou passage en filière CAP / BEP<br>☐ Niveau 5 : BEP / CAP<br>☐ Niveau 4 : Brevet Professionnel / Bac Pro ou Bac Technologique / écoles des formations sanitaires ou sociales<br>☐ Niveau 3 : DUT / BTS<br>☐ Niveau 2 : Licence / Master universitaire<br>☐ Niveau 1 : Ingénieur / Master de Grande Ecole / Doctorat
**D.4.1 Your Job**	☐ Executive<br>☐ Employee<br>☐ Worker<br>☐ Craftsman<br>☐ Freelance<br>☐ Unemployed<br>☐ Retired<br>☐ Student<br>☐ Researcher<br>☐ Engineer<br>☐ Teacher/professor<br>☐ Other (specify):<br>______________________

**D.5 Region where you live**	☐ Auvergne - Rhône-Alpes<br>☐ Bourgogne - Franche-Comté<br>☐ Bretagne<br>☐ Corse<br>☐ Centre - Val de Loire (Centre)<br>☐ Grand Est (Alsace, Champagne Ardenne, Lorraine)<br>☐ Hauts de France (Nord Pas-de-Calais - Picardie)<br>☐ Île de France<br>☐ Nouvelle Aquitaine (Aquitaine, Poitou-Charentes, Limousin)<br>☐ Normandie (Basse Normandie and Haute Normandie)<br>☐ Occitanie (Midi-Pyrénées, Languedoc- Roussillon)<br>☐ Pays de la Loire<br>☐ Provence-Alpes-Côte d'Azur

**D.6 The area where you live**	☐ Rural<br>☐ Urban–periphery

**D.7 Your current salary (net monthly salary)**	☐ en dessous de € 1,000<br>☐ € 1,001 - 1,200<br>☐ € 1,201 – 1,500<br>☐ € 1,501– 1,800<br>☐ € 1,801 – 2,000<br>☐ €2,001– 2,300<br>☐ € 2,301– 3,000<br>☐ €3,001– 4,000<br>☐ € 4,000 – 5,000<br>☐ € 5,001 – 6,000<br>☐ € 6,001 – 7,000<br>☐ € 7,001 – 8,000<br>☐ € 8,001 – 9,000<br>☐ 9,001 et plus

**D.8 Your household size**	Number of adults:<br>Number of children (< 18 years):

THANK YOU FOR YOUR PARTICIPATION IN THE SURVEY

You can add comments in the space below:
