# Supplementary material for: A contingent valuation experiment about future particle accelerators at CERN
Source: PLoS One. 2020 Mar 11;15(3):e0229885. doi: 10.1371/journal.pone.0229885 (PMC7065825; doi:10.1371/journal.pone.0229885)
Supplement: S3 File — (PDF) [file pone.0229885.s003.pdf]

SUPPORTING INFORMATION

A contingent valuation experiment about future particle accelerators at CERN

S3 File. Information material

Link to the two-minute movie in French https://videos.cern.ch/record/2292503

Two-page description of CERN (in French and in English).

QU'EST-CE QUE LE CERN ?

Le CERN (Conseil Européen pour la Recherche Nucléaire) est une organisation internationale pour la recherche fondamentale en physique fondée en 1954. L'objectif de cette recherche est la compréhension des phénomènes naturels. Son site se trouve à cheval sur la frontière franco-suisse, près de Genève. Il s'agissait à sa fondation d'une des premières organisations d'envergure européenne. Il compte aujourd'hui 22 Etats membres et 7 Etats membres associés.

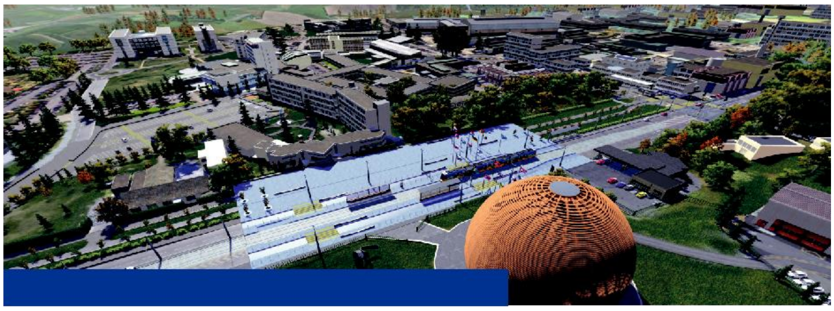

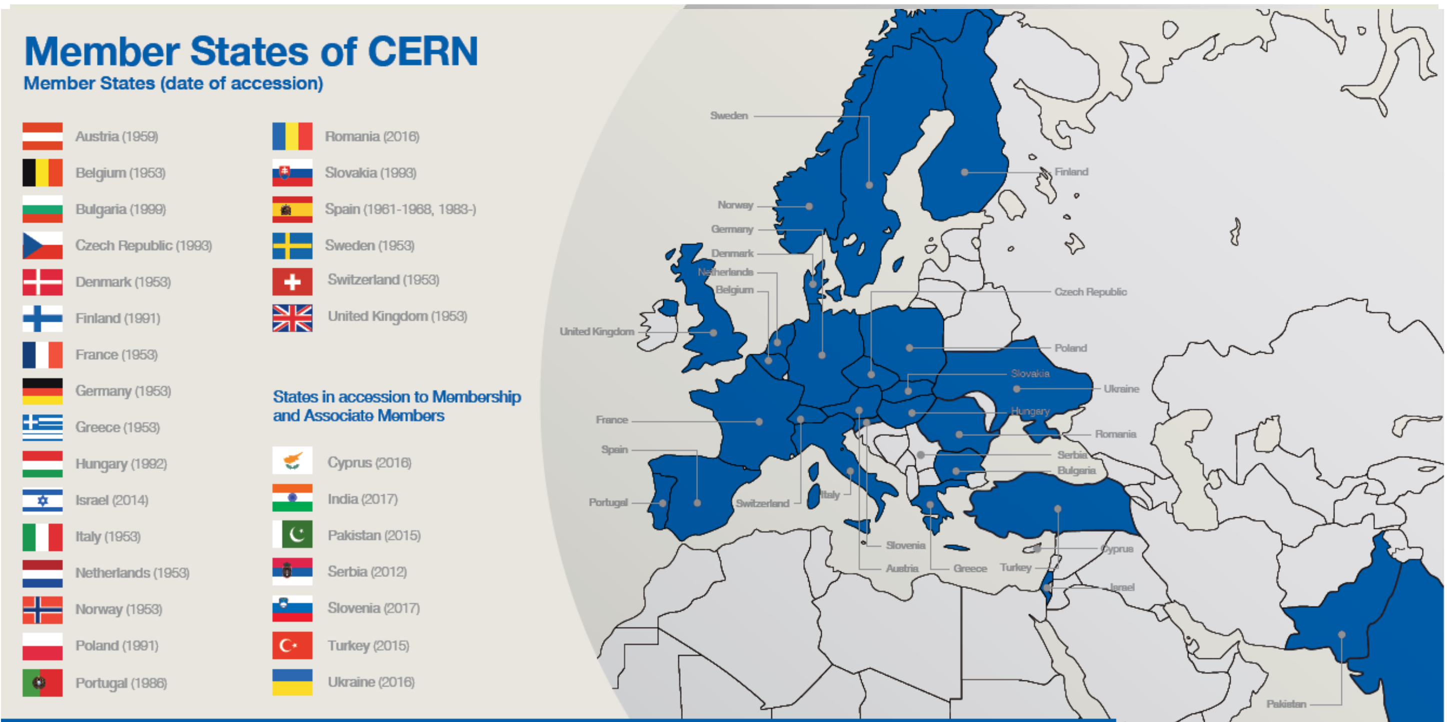

Les Etats membres et les Etats membres associés du CERN, une organisation intergouvernementale de recherche

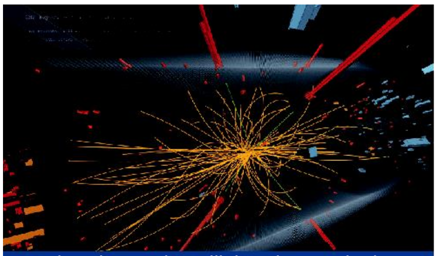

Recherche sur la collision des particules

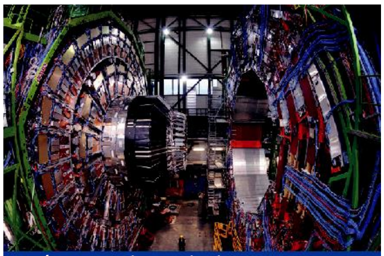

Détecteur de particules

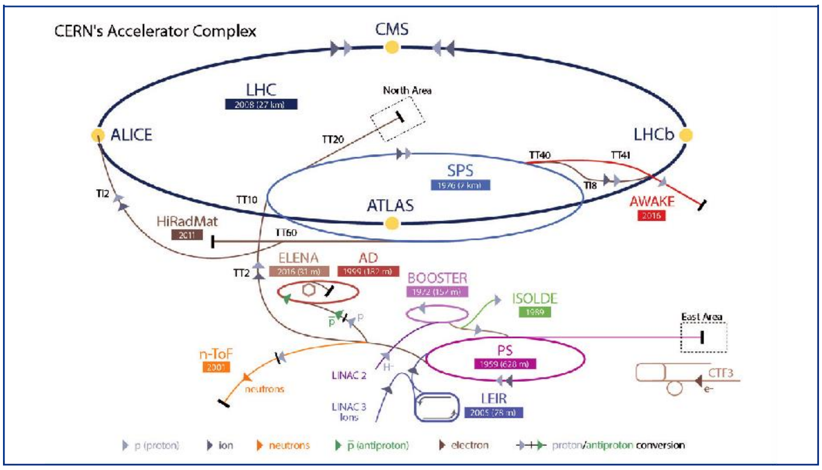

Au CERN, les physiciens explorent les structures fondamentales de l'univers avec de puissants instruments scientifiques et étudient les constituants élémentaires de la matière : les particules fondamentales. Ces particules sont accélérées à une vitesse proche de celle de la lumière et des collisions sont provoquées. Le processus donne aux physiciens des indices sur la façon dont les particules interagissent et permet d'aller plus loin dans la compréhension des lois fondamentales de la nature.

En l'espace de 60 ans, c'est tout un ensemble d'accélérateurs de particules interconnectés qui a été construit. Le Grand Collisionneur de Hadrons (le « Large Hadron Collider » ou « LHC ») est à ce jour le plus grand et le plus puissant accélérateur de particules au monde. Il se trouve dans un tunnel circulaire long de 27 kilomètres, situé environ 100 mètres sous terre. En 2012, il confirme l'existence du boson de Higgs, un maillon essentiel pour la résolution d'un problème majeur de la physique.

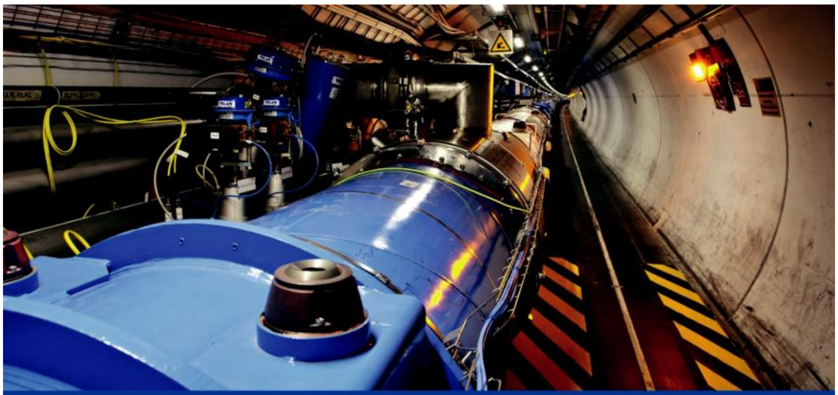

Le LHC, le plus grand et le plus puissant accélérateur de particules au monde.

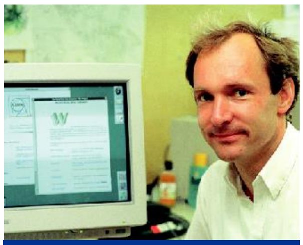

Tim Berners-Lee,
inventeur du WWW

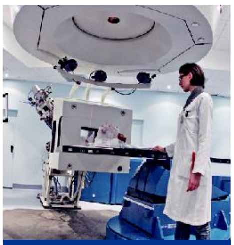

Le traitement
du cancer

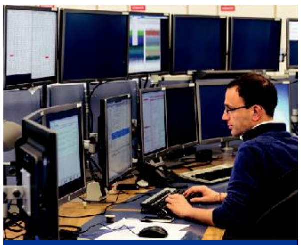

Logiciels (système
de contrôle industriel)

Plus de 12 000 scientifiques, ingénieurs, techniciens, étudiants et prestataires de presque 80 nationalités différentes collaborent sur le site du CERN. De par le monde, un nombre en-core plus conséquent de scientifiques étudie les données récoltées par les accélérateurs de particules grâce à une infrastructure complète dédiée au partage de données et au calcul.

Plusieurs technologies issues du CERN ont trouvé des applications dans d'autres domaines. Le World Wide Web, qui fut inventé au CERN en 1989, est un exemple. Le traitement du cancer avec les accélérateurs de particules en est un autre. Enfin, un grand nombre de logiciels dé-veloppés au CERN ont contribué à rendre plus efficaces les bases de données numériques, à améliorer le contrôle des sites industriels ou à accélérer et sécuriser l'accès à des volumes de données de plus en plus grands.

Le CERN participe aussi à des projets scientifiques internationaux, au-delà de la physique des particules. Il est impliqué dans le projet UNOSAT pour apporter de l'aide humanitaire via l'imagerie satellite. C'est également le cas du projet MEDICIS-PROMED pour développer des éléments chimiques spéciaux pour l'imagerie médicale et du projet SESAME qui entend construire un accélérateur de particules pour la formation aux technologies, la science des matériaux, la biologie, l'archéologie et la chimie.

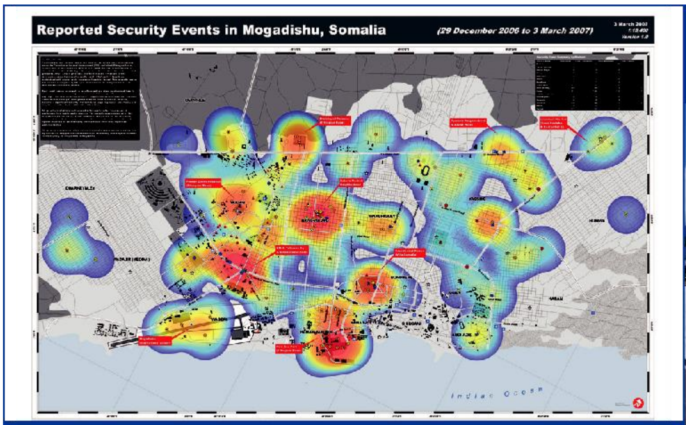

Exemple d'une analyse d'une zone de conflit par imagerie satellite.

Une installation du CERN pour produire des radio-isotopes pour la médecine et la recherche médicale.

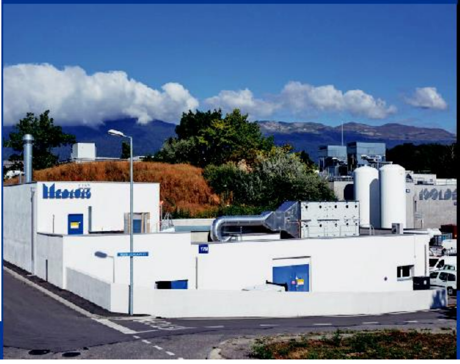

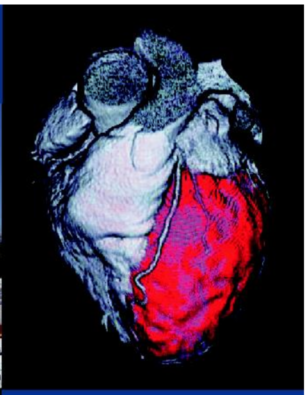

Image du cœur réalisée par émission de positrons.

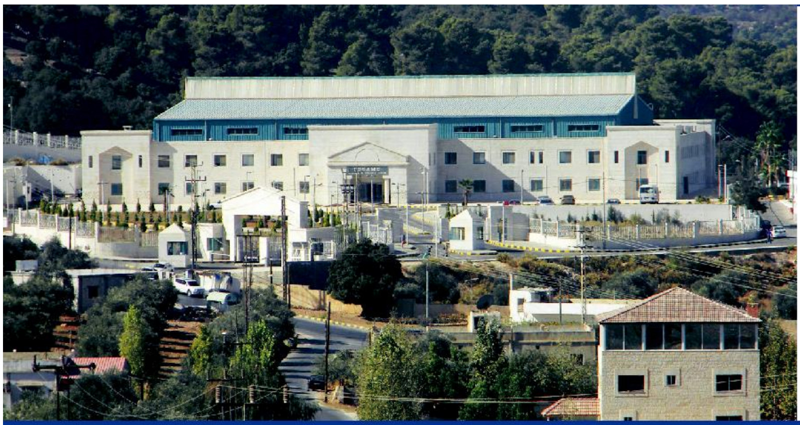

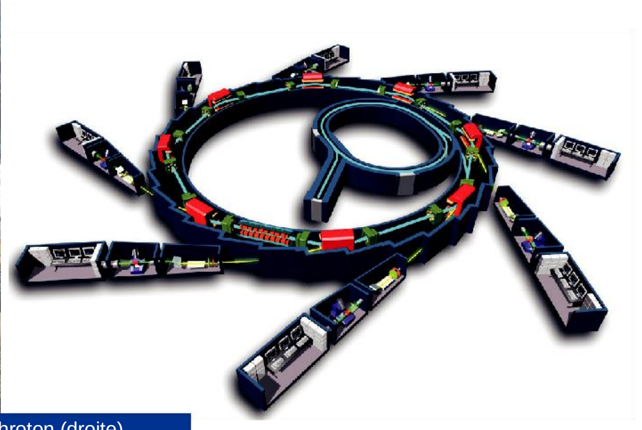

Le bâtiment SESAME à Allan, en Jordanie (gauche) et le schéma du rayonnement synchroton (droite).

Le CERN a fait de son programme éducatif une priorité. Près de 600 étudiants et stagiaires du monde entier arrivent chaque année pour participer à des projets internationaux. Le CERN organise de nombreuses sessions de formation et près de 350 cours différents sur le site et dans le monde entier.

WHAT IS CERN ?

CERN (European Organization for

Nuclear Research) **is an international organization for fundamental research in physics founded in 1954.** The objective of its research is the understanding of the origin of the universe. **Its site straddles the Franco-Swiss border near Geneva.** At its foundation, it was one of the first organizations of European scope. Currently, CERN **has 22 member states and 7 associate member states.**

Aerial view of CERN at the French-Swiss border

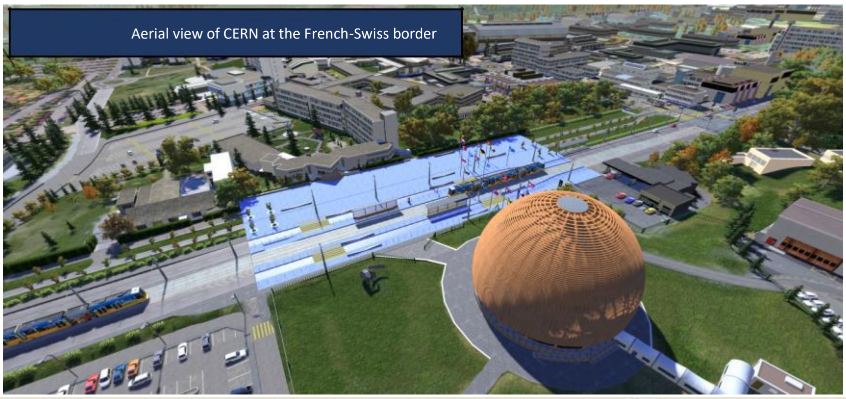

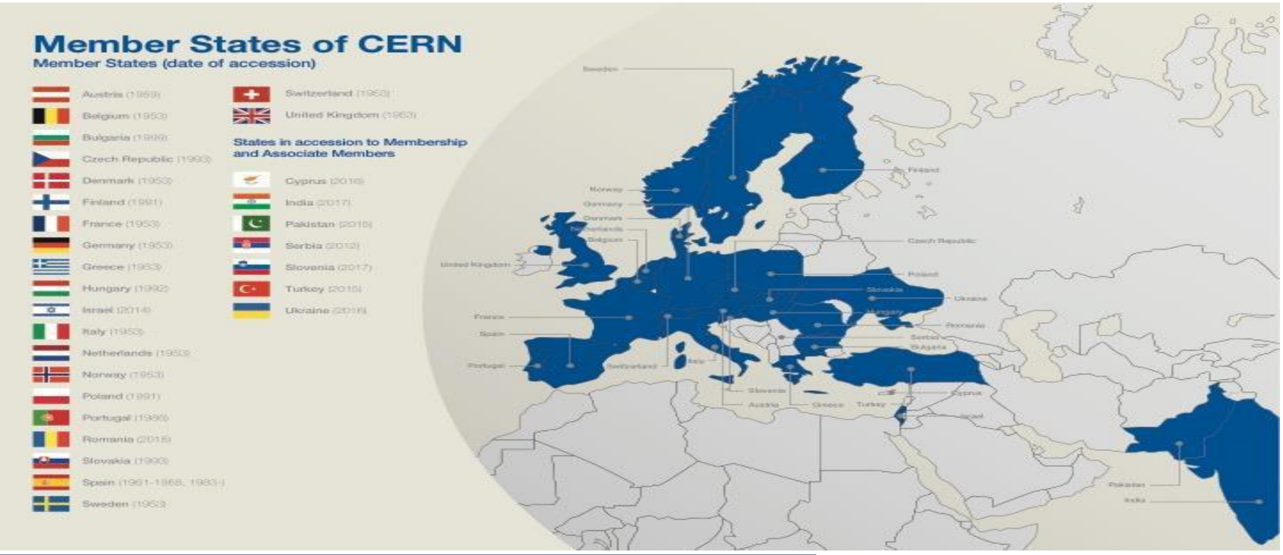

Research on the particle collision

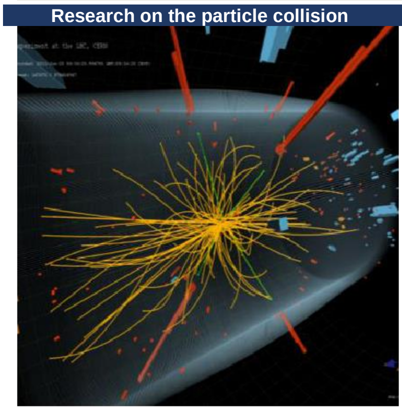

Particle detector

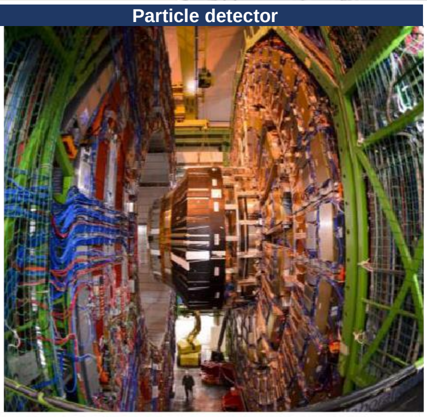

At CERN, **physicists explore the fundamental structures of the universe** with powerful scientific instruments, studying the elementary constituents of matter: the fundamental particles. These particles are accelerated at a speed close to that of light and then collided against their counterparts. The process gives physicists clues about how the particles interact, allowing to go further in understanding the fundamental laws of nature.

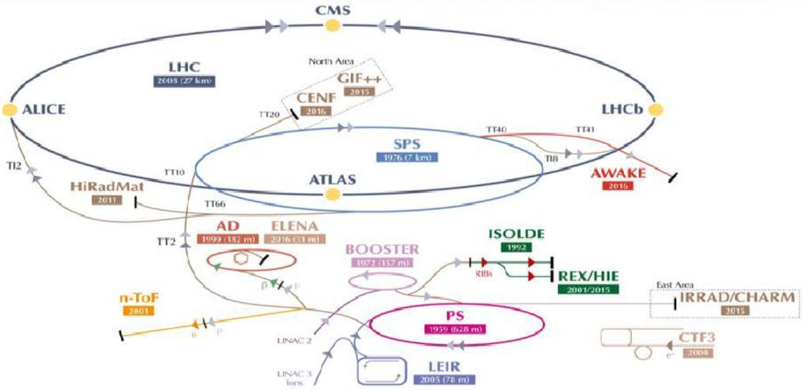

For 60 years, **a whole set of interconnected particle accelerators** has been built**. The Large Hadron Collider (LHC) is currently the largest and most powerful particle accelerator in the world.** It lies in a circular tunnel 27 kilometers long, located about 100 meters underground. In 2012, it confirmed the existence of the Higgs boson, an essential link for the resolution of one of the biggest questions in physics.

System of interconnected particle accelerators

The Large Hadron Collider, the largest and most powerful particle accelerator in the world

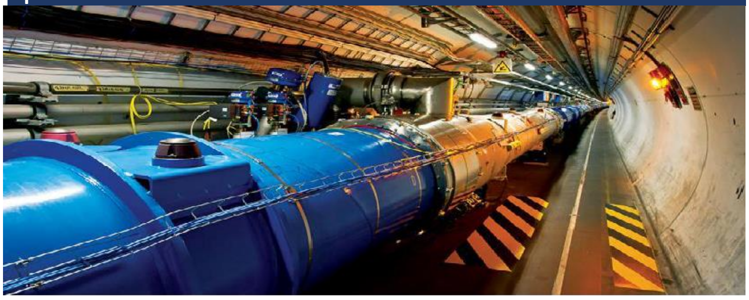

More than 12,000 scientists, engineers, technicians, students and service providers from almost 80 different nationalities work together at CERN. Around the world, more and more scientists are studying data collected by particle accelerators through a comprehensive infrastructure for data sharing and computation.

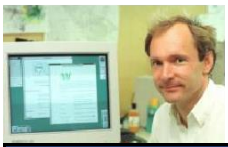

Tim Berners-Lee, the inventor of WWW (World Wide Web)

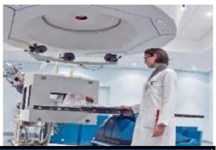

Cancer treatment

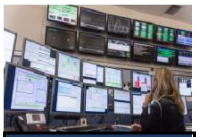

Software (industrial control system)

Several technologies from CERN have found applications in other areas. **The World Wide Web**, invented at CERN in 1989, is an example.

The cancer treatment with particle accelerators is another. Additionally, **a large number of software** developed at CERN has helped make digital databases more efficient, improve the control of industrial sites or speed up and secure access to even-larger volumes of data.

CERN also participates in international scientific projects, beyond particle physics. It is involved in the UNOSAT project to provide humanitarian aid via satellite imagery. This is also the case of the MEDICIS-PROMED project to develop special chemical elements for medical imaging and the SESAME project, which intends to build a particle accelerator for training in technology, materials science, biology, archaeology and chemistry.

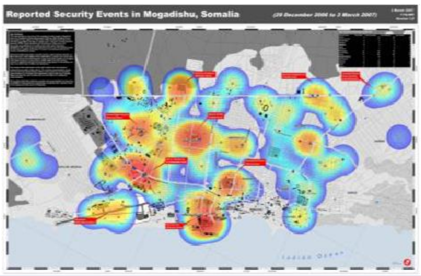

Analysis of a conflict zone by satellite imagery

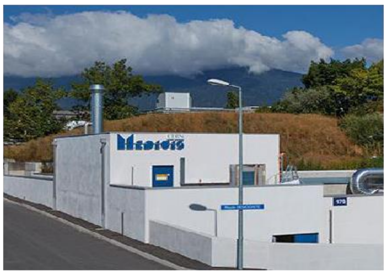

CERN facility to produce radioisotopes for medical research

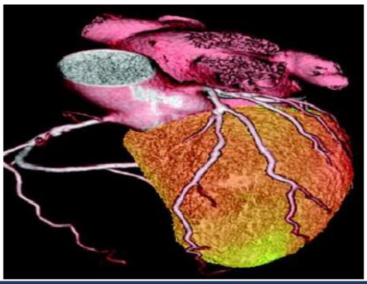

Image of the heart made by positron emissions

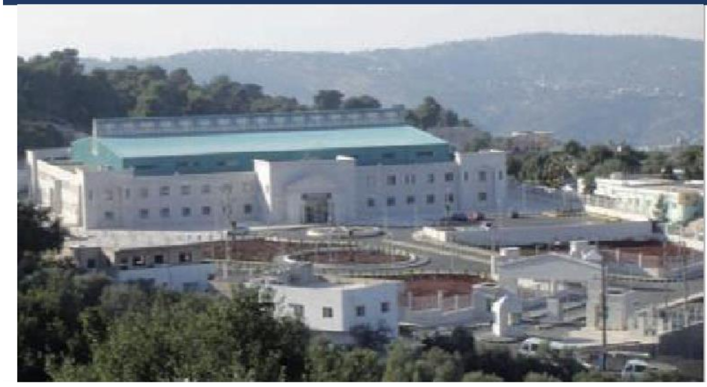

The SESAME building in Allan, Jordan

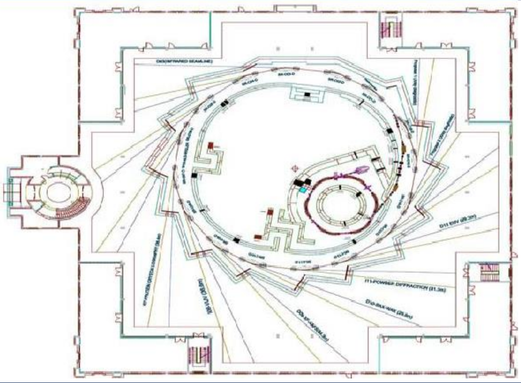

The synchrotron radiation diagram

CERN has made its educational program a priority. **Approximately 600 students and trainees from around the world** arrive each year to participate in international projects. CERN organizes numerous training sessions and around 350 different courses at the site and around the world.
