# Supplementary material for: A contingent valuation experiment about future particle accelerators at CERN
Source: PLoS One. 2020 Mar 11;15(3):e0229885. doi: 10.1371/journal.pone.0229885 (PMC7065825; doi:10.1371/journal.pone.0229885)
Supplement: S4 File — (PDF) [file pone.0229885.s004.pdf]

SUPPORTING INFORMATION

A contingent valuation experiment about future particle accelerators at CERN

S4 File. The informed consent

Please, read carefully this document before participating in the survey.

Dear Sir or Madam,

We invite you to participate in an inquiry to find out your opinion on the value of the research activities carried out at CERN (European Organization for Nuclear Research), an international organization whose convention was ratified in 1954 and amended in 1971.

Eumetra MR S.r.l. and the University of Milan (Italy) carry out the survey on behalf of CERN. The Ethics Committee of the University of Milan approved it on October 31th, 2018.

You are one of the people selected to form a sample of the French population and invited to express themselves on this subject. Your participation is therefore greatly appreciated and your sincere opinion can help make decisions about strengthening research activities at CERN. Please note that due to possible conflicts of interest, having or having had professional relations with CERN (contract as employee, student or associate staff member, contractual relations, activities at CERN, contractors or subcontractors) does not allow you to participate in this survey.

During this survey, CERN's research activities are introduced by means of a video (approximately two minutes) and a short document (two pages). Then, you are asked to express your opinion on the research activities carried out at CERN and to indicate whether you would be ready to support new investments for this type of research. At the end of the survey, you are also asked some additional information, such as region of residence, age group, your income class. This will allow us to read the results both in total and in relation with the main socio-demographic parameters.

The survey fully protects your privacy and guarantees the confidentiality of the information provided. Click on the link to access more information about privacy:

https://www.cnil.fr/sites/default/files/typo/document/Act78-17VA.pdf

Should you have any questions about your rights as a participant in this survey, or should you wish to obtain additional information or express any concerns about this survey, please contact Mr. XX, from Eumetra MR S.r.l. (email address: XX)

In order to participate in the survey, please agree with the statements below.

i. I declare that I have no connection with CERN (for example, former staff member, and person having business with CERN, contractor or equivalent).

◻ YES

ii. I agree to participate in this survey. I understand the purpose and nature of this investigation and I participate voluntarily. I understand that I may stop participating in this survey at any time without penalty or consequence.

UNIVERSITÀ DEGLI STUDI DI MILANO

□ YES

iii. I understand the relevance of the results of this survey to the public. I authorize the use of the data of this interview in an anonymous and consolidated form, in accordance with the French regulations in force on information technology, data files and civil liberties.

□ YES

iv. I understand the relevance of this survey for CERN and for its investments in future research activities. I therefore undertake to provide honest and sincere answers.

□ YES

By signing and returning this form, you confirm that you have read the document and agree to participate in the survey.

Place and date : Signature
