# Supplementary material for: A contingent valuation experiment about future particle accelerators at CERN
Source: PLoS One. 2020 Mar 11;15(3):e0229885. doi: 10.1371/journal.pone.0229885 (PMC7065825; doi:10.1371/journal.pone.0229885)
Supplement: S5 File — (PDF) [file pone.0229885.s005.pdf]

SUPPORTING INFORMATION
A contingent valuation experiment about future particle accelerators at CERN

S5 File. Respondents' socioeconomic traits, personal interests and perceptions of CERN

Table A. French population vs sample (N=1,005).
Percentage distribution according to age, gender, education, income, and region of residence.

Variable	Population (%)	Sample (%)
Gender		
Female	51.0	51.0
Male	49.0	49.0
Age (years)		
16-24	14.7	14.7
25-34	17.0	17.0
35-44	18.1	18.0
45-54	19.0	18.9
55-64	17.5	17.5
65-74	13.7	13.9
Education level		
Low	28.9	28.5
Medium	41.6	41.0
High	30.6	30.5
Per-capita annual net income		
Less than EUR 15,000	21.4	29.1
EUR 15,000-22,000	28.7	29.3
EUR 22,000-28,000	23.2	17.3
More than EUR 28,000	26.7	24.2
Region of residence		
Île de France	19.2	19.6
Auvergne-Rhône-Alpes	12.2	12.5
Provence-Alpes-Côte d'Azur	7.8	6.8
Nord - Pas-de-Calais	6.4	6.8
Pays de la Loire	5.7	6.5
Aquitaine	5.3	4.6
Bretagne	5.1	5.3
Midi-Pyrénées	4.7	6.1
Languedoc-Roussillon	4.4	5.7
Centre (FR)	4.0	3.6
Lorraine	3.7	2.9
Picardie	3.0	2.7
Alsace	3.0	3.5
Haute-Normandie	2.9	3.9
Poitou-Charentes	2.8	2.3
Bourgogne	2.5	2.1
Basse-Normandie	2.3	1.9
Champagne-Ardenne	2.1	1.4
Franche-Comté	1.8	1.3
Limousin	1.1	0.8

Note. The classification of the education level is as follows. Low includes the French educational levels "Niveau (hereafter "level") 6, 5bis, and 5". Level 6 refers to who left education in the first three years of secondary education or prevocational training courses (e.g. primary education certificate - CEP), prevocational classes (CPPN) and preparatory apprenticeship classes CPA) in one year. Level 5bis includes those who left education in the 4th year of general secondary education, the 3rd or 4th year of a technical secondary course

or shorter secondary education courses before the final year; while level 5 refers to those who left education having completed the final year of a short vocational course or dropped out of schooling during general secondary education before the final year. Medium education includes "Level 4"; that is those who left education having reached the final year of general secondary education or who dropped out if education after the Baccalaureate (BAC) without reaching Level 3. High education level includes: "Level 3, Level 2, and Level 1". People with level 3 of education are those who left having obtained a "BAC+2" qualification, and specifically, successfully completed two years of education after the BAC such as the technological university diploma (DUT), higher technical certificate (BTS), diploma of general university studies (DEUG). Level 2 and 1 include those who obtained a bachelor's degree-level or postgraduate qualification or a diploma awarded by a Grandes Écoles, i.e. higher education prestigious establishments that are outside the main framework of the French public university system.

Table B. Additional demographics of the sample (*N* =1,005).

Variable	(%)
Employment status	
Student	9.7
Employed	59.5
Retired	18.1
Unemployed	12.7
Area	
Urban-periphery	60.2
Rural	39.8
Family size	
1 member	21.6
2-3 members	51.4
More than 3 members	27.0

Figure A. Interests of respondents by topic (N=1,005). .

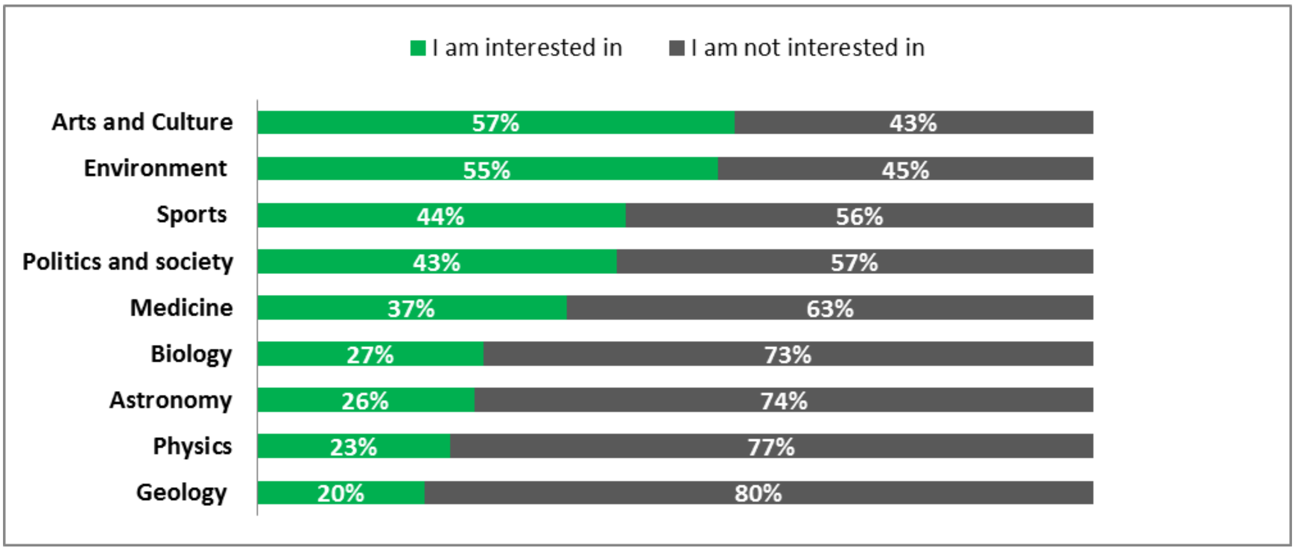

Note: Question A.1. To what extent are you interested in the following topics? Please answer each question by ticking you preferred option. Original Scale: 1 = not at all; 2 = very little; 3=little; 4= enough; 5 = much. In the figure, "I am interested in" groups 4 and 5; and "I am not interested in" groups: 1, 2, and 3. English translation.

Figure B. Importance of scientific research in general (N=1,005).

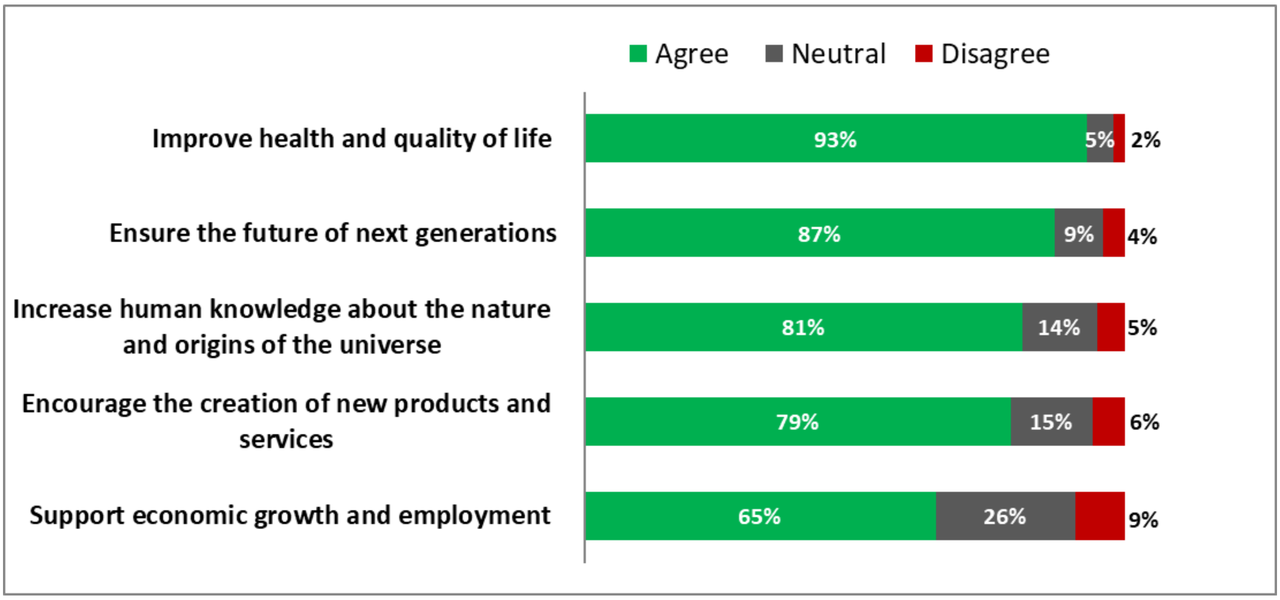

Note: Question A.3. Please, answer each question by ticking your preferred option. Scientific research is important to … Original Scale: 1 = strongly disagree; 2 = disagree; 3=neutral; 4= agree; 5 = strongly agree. In the figure "Agree" groups 4 and 5; and "Disagree" 1 and 2. English translation.

Figure C. Respondents' perceptions about CERN (N=1,005).

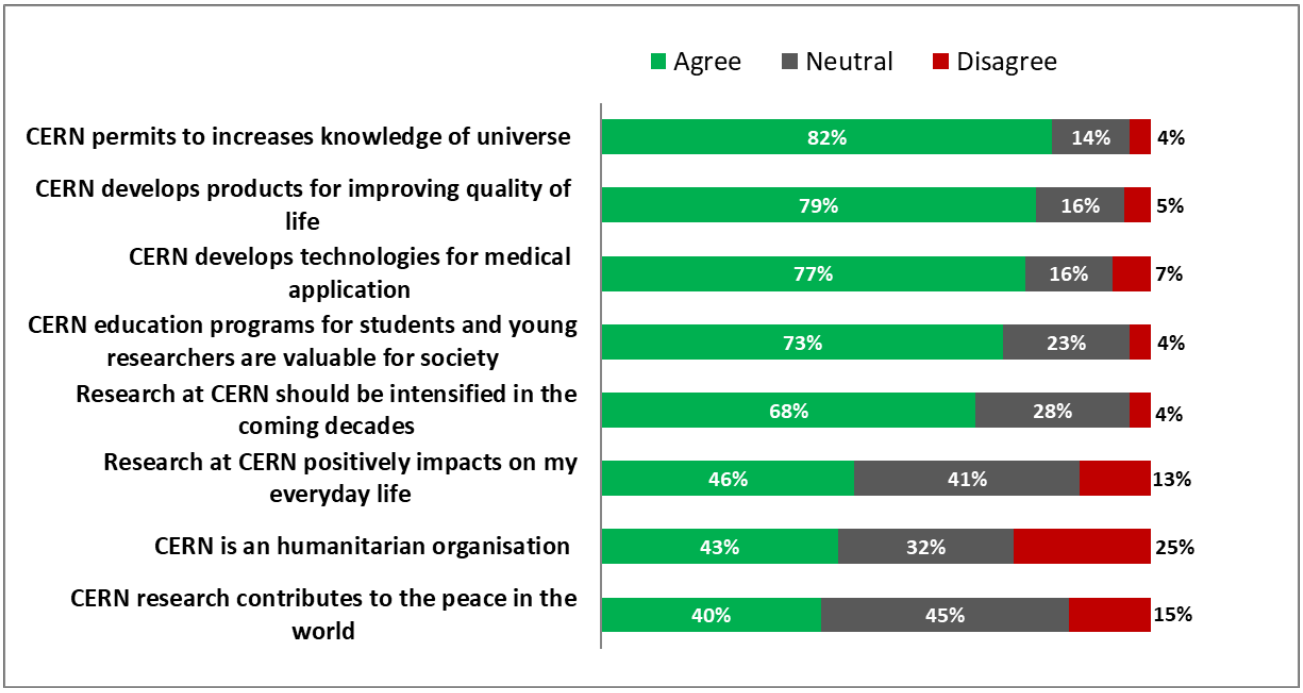

Note: Question B.4 and B.6. For each statement, choose your level of agreement. Original Scale: 1 = strongly disagree; 2 = disagree; 3=neutral; 4= agree; 5 = strongly agree. In the figure "Agree" groups 4 and 5; and "Disagree" 1 and 2. English translation.

Table C. Perceptions of respondents about CERN (N= 1,005).

N indicates the number of times respondents cited the corresponding words or expressions.

**Question B.1. Which aspect of CERN do you appreciate?**	N	**Question B.1. Which aspect of CERN do you not appreciate?**	N
“Research”, “scientific research”, “research laboratory”	294	Nothing	576
Research about the universe, big-bang, research about particle physics, particles accelerator, nuclear research, physics research	222	Too complex / difficult subject	68
Medical research	180	Lack of security / Too dangerous	67
Development and innovation	145	Lack of communication about activities / Lack of information	45
International institution, European institution, major institution, famous, serious organization, organization located in Switzerland / near the French-Swiss border	139	Nuclear	43
Possibility of a better future, Important for future generations	25	Excessive costs	37
Other	84	Useless experiences / No concrete application / Not needed	16
Nothing	70	Other	54
Everything	49	Everything	10
No answer	94	No answer	115
**Total**	**1,302**	**Total**	**1,031**

Note. Open-ended questions. Both questions were asked after the respondents’ exposure to the information material (English translation). When possible, items were aggregated. For instance, expressions such as “research”, “scientific research”, “research laboratory” denote perceptions of generic research, therefore, they were counted together: these expressions were written down 294 times.

Figure D. Importance of scientific research at CERN (N= 1,005).

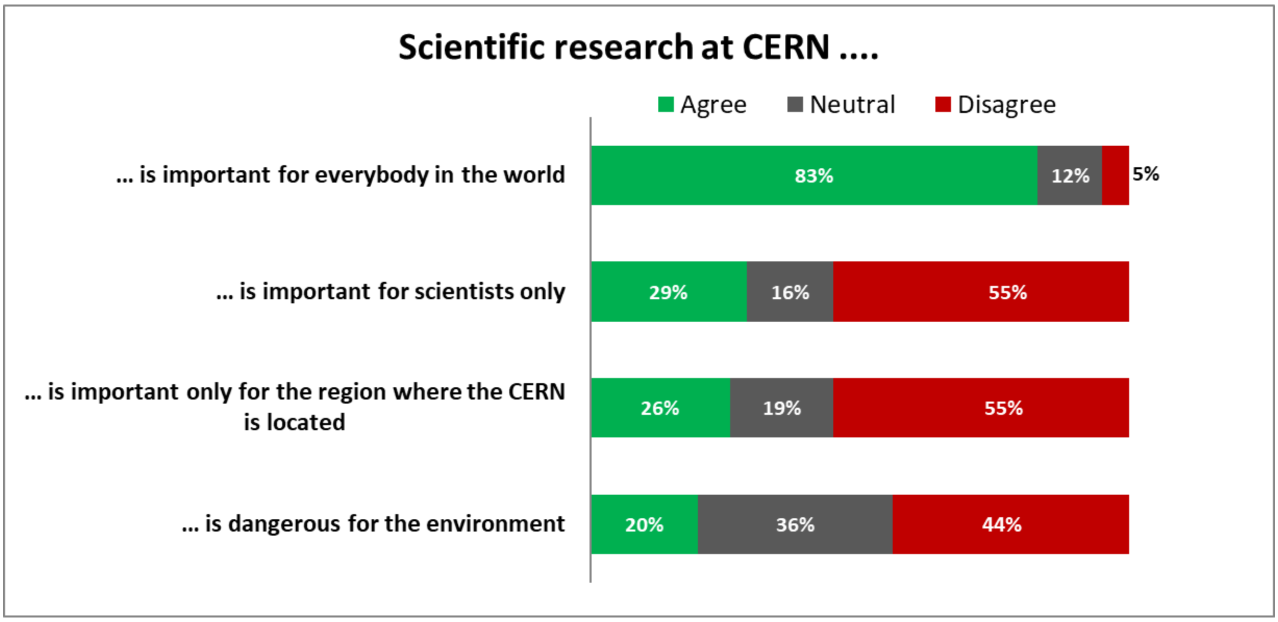

Note: Question B.5. For each statement, choose your level of agreement. Original Scale: 1 = strongly disagree; 2 = disagree; 3=neutral; 4= agree; 5 = strongly agree. In the figure “Agree” groups 4 and 5; and “Disagree” 1 and 2. English translation

Table D. Frequency of the stated reasons why respondents were either not willing to pay (WTP =0) or were willing to pay (WTP>0) for new investments in particle physics research at CERN (N= 1,005).

N indicates the number of people who ticked that option.

**Question C.4 What is the main reason you would pay zero? Please, choose at most two options.**	N	**Question C.6. Why would you pay your amount? Please, choose at most two options**.	N
Currently, I cannot afford this expense	275	I think that CERN's activities justify at least the bid amount asked	252
I prefer to spend my money for other things	175	The whole world benefits from CERN activities	183
I think that my money should be used to fund other research activities	52	We should always support this type of research, even if the results do not necessarily lead to any concrete application	118
I do not understand the purpose of CERN's scientific research, therefore I do not want to contribute	45	Research permits companies to develop new products and services	114
Only people that directly benefiting from CERN's research activities should pay for it	35	France benefits from CERN activities	84
I am against government-funded programs	20	Through this research, the competences/skills of all people involved in the projects will grow	71
I am against international organizations	9	Europe benefits from CERN activities	30
Other reasons	41	Other reasons	10
