# Supplementary material for: A contingent valuation experiment about future particle accelerators at CERN
Source: PLoS One. 2020 Mar 11;15(3):e0229885. doi: 10.1371/journal.pone.0229885 (PMC7065825; doi:10.1371/journal.pone.0229885)
Supplement: S6 File — (PDF) [file pone.0229885.s006.pdf]

SUPPORTING INFORMATION
A contingent valuation experiment about future particle accelerators at CERN

S6 File. Supplementary analysis

The guidance for SP studies [1] recommends ex-post robustness checks to enhance the credibility and transparency of empirical results associated with a CV experiment. We check two sets of assumptions we made during data analysis. The first set regards the sample trim we applied to estimate the mean WTP by excluding protests answers and outliers; the second set regards the pre-treatment of the variables entering in the econometric analysis.

29 respondents who are “against government-funded programs” or “international organisations” are identified as protestors and therefore excluded from the WTP calculation. However, one may argue that they are legitimate answers to signal that further investments would be worth nothing to them and hence should be taken on board. In addition to protestors, 12 respondents are labelled as outliers, because of having a ratio between the stated maximum WTP and their income higher than 0.5%. Again, one may argue that such a threshold is arbitrary and that may there exist generous people willing to pay much for investments at CERN. Accordingly, we test the impact of our choices by calculating the mean WTP from three different (sub-) samples and comparing the result with our reference mean WTP as presented in the main text. Table A reports the outcomes of this test. For each model - the DBDC-CV, Spike, and the non-parametric sample mean of the stated maximum WTP – we estimate the mean WTP from:

- the entire sample (*N=1,005*) where both protestors and outliers are included;
- the trimmed sample at the upper tail of the WTP distribution (*N = 993*) where protestors are included and outliers excluded;
- the trimmed sample at the lower tail of the WTP distribution (*N = 977*) where protestors are excluded and outliers included.

Column 3 reports the mean WTP estimated with a specification that only includes the bid variable as independent variable (no other covariates are included); while standard errors and confidence intervals are shown in Column 4 and 5, respectively. The null hypothesis that our reference mean WTP (*N= 965*) is statistically equal to the mean WTP as estimated in Column 3 is tested in Column 6. The first finding is that whatever the model and sample considered, the mean WTP is always statistically different from zero and higher of the actual contribution French citizens pay to CERN through taxes. Secondly, apart from the DBDC-CV model which does not properly fit the asymmetry of the WTP distribution in the data, both Pearson's chi-square tests (when considering the spike-model) and t-tests (for the maximum WTP) do not reject the null hypothesis of mean equality. This suggests that the asymmetry of the WTP, whence perhaps half of the population does not want to pay, is not driven by ‘protest-bids’, but rather than by the fact that research in particle physics at CERN does not belong to the utility function. In contrast, the mean WTP is statistically higher than the reference one when outliers are included. It turned to be EUR 19.06 per person per year in the spike model; while the sample mean of stated maximum WTP is EUR 17.28 per person per year.

Table A. Robustness checks

Model	Obs	Mean WTP	Std. err	CI[a]	Testing H0: Mean WTP trimmed sample (*N=965*) – Mean WTP = 0
(1)	(2)	(3)	(4)	(5)	(6)
**Double bounded dichotomous choice (DBDC-CV)**					
Entire sample	1,005	2.87*	1.79	[-0.64; 6.39]	Chi2 = *13.12****
Including protest answers; excluding outliers	993	2.79*	1.67	[-0.48; 6.06]	Chi2 = *22.71****
Excluding protest answers; including outliers	977	4.16***	1.77	[0.68; 7.64]	Chi2 = *1.10*
**Spike**					
Entire sample	1,005	18.55***	1.26	[16.09; 21.02]	Chi2 =*9.05****
Including protest answers; excluding outliers	993	16.47***	1.12	[14.27; 18.68]	Chi2 =*1.46*
Excluding protest answers; including outliers	977	19.06***	1.29	[16.52; 21.59]	Chi2 = *8.73****
**Sample mean of the stated maximum WTP**					
Entire sample	1,005	16.80***	1.80	[13.28; 20.32][b]	t= *1.87**
Including protest answers; excluding outliers	993	13.09***	0.94	[11.24; 14.94][b]	t = *-0.4064*
Excluding protest answers; including outliers	977	17.28***	1.78	[13.80; 20.77][b]	t=*2.0793***

Note. ***, **, * denote significance at the 1%, 5%, 10% level respectively. [a]Confidence intervals. [b]Bootstrapped confidence intervals with 1,000 replications.

The second set of robustness checks regards the pre-treatment of the variables entering in the econometric analysis as shown in Table 2 in the main text. We present results where we decided to collapse variables from the list shown in Table A. For instance, in the econometric analysis we used the binary variable “Age (<35)” instead of using the original six categories of age. Similarly, we used the binary variable “Family Size (>3 members)” instead of the original three categories of family size. Yet, we constructed the variable “Scientific interest” as a composite categorical variable instead of testing the scientific interests in medicine, biology, physics, astronomy, physics, and geology separately. Our choices were based on reflections that the understanding of “youth” across European countries keeps the upper age limit mainly towards 35 when some policy areas are considered (employment, housing, social protection, etc…) [2]; that a family size of three members is higher than the average size of French households, which is 2.38 persons per household (http://www.oecd.org/social/soc/doingbetterforfamilies.htm) and that interests in scientific disciplines could be aggregated as to differentiate them from interests in arts and cultures in general, environment and so on. However, we want to see to what extent the use of the original definition of variables will bring new or different results.

No age effects emerge from the DBDC-CV model, in contrast the spike model suggests that older people aged between 45-54; 55-64; 65-74 would be willing to pay more with respect to younger

people aged between 16-24, used as reference category. However, this result is not robust in the sense that age loses its predicting power when education, awareness, and scientific interests are jointly plugged into the same specification.

No new evidence arises regarding family size with respect the above analysis: while showing a negative and statistically significative effects in the DBDC-CV model (the more numerous the family, the lower the WTP); the impact of this variable is statistically not different from zero in the spike model.

As far as interests are concerned, having an interest in physics has a positive and statistically significant impact on the WTP for particle accelerators investments at CERN with respect to not being interested in physics.

The single bounded dichotomous choice (SBDC-CV) model

The DBDC-CV has the great power of returning more efficient WTP estimators than the SBDC-CV estimator, but it risks anchoring [3] and to violate incentive compatibility, preventing respondents to truthfully reveal any private information they are asked for [4]. Therefore, we repeat our analysis based just on the first willingness to pay question (bid). We do this both parametrically and non-parametrically.

Starting from Eq. 3 in the main text, we estimated a probit model and whatever the specification used (including covariates or not) the mean WTP was always negative, never statistically different from zero and with a standard deviation larger than the mean. No relevant differences emerged when estimating a logit model. The issue of addressing negative WTP in dichotomous choice CV is largely discussed in the CV literature [1, 5, 6] and the topic goes beyond the scope of this research. One of the causes may rely on the wrong distributional assumption of normality (or logistic) underlying the parametric model. Hence, for comparison, we also examined the performance of the nonparametric Turnbull approach [7]. It is based on the probability or frequency information for "yes" responses in a DC format and assumes non-negative values. Table B shows the distribution of "yes" according the first bid and the survivor function associated with "yes" answers. The Turnbull lower bound estimator yields a mean WTP of EUR 16.08 per person per year statistically different from zero at 1% level (*p-value* < 0.01), which is very close to the mean WTP obtained through the spike model. It was estimated using the Turnbull ado-file for Stata provided by Azevedo [8]

Table B. The single bounded dichotomous choice SBDC-CV model: results from the Turnbull estimator (*N* = 965)

Bid (EUR)	N of respondents in the sub-sample with the same bid	% of "yes" in the sub-sample (survivor function)	Lower bound mean WTP per year per person
3	194	52.06	EUR 16.08***
5	198	42.42	
10	195	40.00	
30	192	33.33	
50	186	28.49	

Note. ***, **, * denote significance at the 1%, 5%, 10% level respectively. Estimation is net of protests answers and outliers.

References

1. Johnston RJ, Boyle KJ, Adamowicz W, Bennett J, Brouwer R, Cameron TA, et al. Contemporary guidance for stated preference studies. Journal of the Association of Environmental and Resource Economists**.** 2017; 4: 319-405. https://doi.org/10.1086/691697
2. Youth Partnership. Defining Youth in Contemporary National Legal and Policy Frameworks Across Europe. Technical Report.Youth Partnership, partnership between European Commission and the Council of Europe in the field of youth. Brussels, 2016. Available from https://pjpeu.coe.int/documents/1017981/1668203/Analytical+paper+Youth+Age+Bojana+Perovic+4.4.16.pdf/eb59c5e2-45d8-4e70-b672-f8de0a5ca08c
3. Belyaev Yu K, Kriström B. Analysis of survey data containing rounded censoring intervals. Informatics and Applications. 2015; 3: 2-16 https://doi.org/10.14357/19922264150301
4. Carson RT, Groves T, List JA. Consequentiality: A theoretical and experimental exploration of a single binary choice. Journal of the Association of Environmental and Resource Economists. 2014; 1:171-207 https://www.journals.uchicago.edu/doi/full/10.1086/676450
5. Kriström B. Spike models in contingent valuation. American Journal of Agricultural Economics. 1997; 79: 1013-1023 https://doi.org/10.2307/1244440
6. Bohara AK, Kerkvliet J, Berrens RP. Addressing negative willingness to pay in dichotomous choice contingent valuation. Environmental and Resource Economics. 2001; 3:173-195 https://doi.org/10.1023/A:1012642902910
7. Haab T, McConnell K. Alternative methods for handling negative willingness to pay in referendum models. Journal of Environmental Economics and Management. 1997; 32: 251-270 https://doi.org/10.1006/jeem.1996.0968
8. Azevedo JP. Turnbull: Stata module to estimate the Turnbull empirical distribution estimator of willingness to pay. Statistical Software Components S457125, Boston College Department of Economics. 2010. Available from https://ideas.repec.org/c/boc/bocode/s457125.html
