# Supplementary material for: A contingent valuation experiment about future particle accelerators at CERN
Source: PLoS One. 2020 Mar 11;15(3):e0229885. doi: 10.1371/journal.pone.0229885 (PMC7065825; doi:10.1371/journal.pone.0229885)
Supplement: S7 File — (PDF) [file pone.0229885.s007.pdf]

**SUPPORTING INFORMATION**

**A contingent valuation experiment about future particle accelerators at CERN**

**S7 File. Drivers of the WTP: econometric analysis**

**Table A**. The double bounded dichotomous choice (DBDC-CV) model: results (*N* = 965)

| Variable | (1) | (2) | (3) | (4) | (5) | (6) | (7) |
|---|---|---|---|---|---|---|---|
| Constant | 3.97 (1.65)** | -16.46 (3.70)*** | -16.01 (4.48)*** | -21.81 (5.67)*** | -25.82 (5.40)*** | -26.46 (5.74)*** | -46.61 (7.08)*** |
| Income | | 8.67 (1.33)*** | 7.07 (1.47)*** | 8.35 (1.62)*** | 7.58 (1.62)*** | 6.98 (1.60)*** | 6.60 (1.60)*** |
| Male | | | 3.95 (3.13) | 3.46 (3.15) | 1.61 (3.17) | 1.59 (3.13) | 3.19 (3.13) |
| Age (< 35) | | | -0.65 (3.38) | -1.47 (3.85) | 0.52 (3.87) | 0.93 (3.83) | 2.41 (3.83) |
| Education | | | | | | | |
| *Medium* | | | -2.28 (3.73) | -2.12 (3.76) | -2.86 (3.76) | -3.40 (3.72) | -2.66 (3.73) |
| *High* | | | 8.55 (3.99)** | 8.06 (4.02)** | 6.83 (4.03)* | 4.08 (4.00) | 4.30 (3.99) |
| Occupation status | | | | | | | |
| *Student* | | | | 16.60 (6.67)** | 16.18 (6.65)** | 12.14 (6.58)** | 9.39 (6.58) |
| *Employed* | | | | 3.70 (5.00) | 4.76 (5.00) | 3.81 (4.94) | 3.24 (4.93) |
| *Retired* | | | | 8.66 (5.86) | 9.14 (5.86) | 7.64 (5.79) | 5.49 (5.79) |
| Family Size (*>3 members)* | | | | -7.65 (3.52)** | -7.08 (3.50)** | -9.53 (3.51)*** | -10.54 (3.50)*** |
| Rhône-Alpes | | | -3.54 (4.77) | -3.26 (4.77) | -3.99 (4.77) | -4.11 (4.72) | -2.44 (4.73) |
| Awareness of CERN | | | | | 13.25 (3.09)*** | 9.03 (3.12)*** | 5.39 (3.14)* |
| Scientific interest | | | | | | 5.10 (0.91)*** | 4.21 (0.91)*** |
| CERN permits an increase in knowledge of universe | | | | | | | 11.85 (3.66)*** |
| The research activity at CERN should increase in the coming decades | | | | | | | 18.55 (3.66)*** |
| | | | | | | | |
| Sigma | 40.24 (1.88)*** | 38.97 (1.81)*** | 38.51 (1.79)*** | 38.35 (1.78)*** | 38.07 (1.77)*** | 37.38 (1.73)*** | 36.81 (1.69)*** |
| Observations | 965 | 965 | 965 | 965 | 965 | 965 | 965 |
| Log- likelihood | -1,281.54 | -1,259.36 | -1,252.81 | -1,246.14 | -1,236.72 | -1,220.64 | -1,195.44 |
| Likelihood ratio test | - | 42.72*** | 54.93*** | 65.89*** | 80.84*** | 108.59*** | 136.34*** |
| AIC | 2567.1 | 2576.8 | 2521.6 | 2516.3 | 2499.5 | 2469.3 | 2422.9 |
| BIC | 2576.8 | 2539.3 | 2560. 6 | 2574.7 | 2562.8 | 2537.6 | 2500.8 |

*Note.* Standard errors in parenthesis. ***, **, * denote significance at the 1%, 5%, 10% level respectively. *Male*: missing category is female; *Age (<35)*: omitted category is age > 35; *Education*: omitted category is low; *Occupation status*: omitted category is unemployed; *Family size (>3 members)*: omitted category is family size < 3 members

**Table B**. The spike model: results (*N* = 965)

| Variable | (1) | (2) | (3) | (4) | (5) | (6) | (7) | (8) |
|---|---|---|---|---|---|---|---|---|
| Bid | 0.04 (0.00)*** | 0.02 (0.00)*** | 0.02 (0.00)*** | 0.02 (0.00)*** | 0.02 (0.00)*** | 0.02 (0.00)*** | 0.02 (0.00)*** | 0.02 (0.00)*** |
| Constant | -0.02 (0.06) | 0.03 (0.06) | 0.05 (0.06) | 0.05 (0.06) | 0.05 (0.06) | 0.05 (0.06) | 0.05 (0.06) | 0.05 (0.06) |
| Income | | 0.22 (0.03)*** | 0.14 (0.04)*** | 0.07 (0.05)* | 0.05 (0.06) | 0.08 (0.05)* | 0.05 (0.06) | 0.06 (0.06) |
| Male | | | 0.14 (0.11) | 0.14 (0.11) | 0.09 (0.11) | 0.15 (0.11) | 0.09 (0.11) | 0.15 (0.11) |
| Age (< 35) | | | 0.49 (0.12)*** | 0.60 (0.16)*** | 0.64 (0.16)*** | 0.60 (0.16)*** | 0.57 (0.16)*** | 0.59 (0.16)*** |
| Education | | | | | | | | |
| *Medium* | | | -0.11 (0.13) | -0.10 (0.13) | -0.13 (0.14) | -0.09 (0.13) | -0.12 (0.14) | -0.09 (0.13) |
| *High* | | | 0.25 (0.13)** | 0.23 (0.14)* | 0.22 (0.13)* | 0.23 (0.14)* | 0.15 (0.14) | 0.25 (0.14)* |
| Occupation status | | | | | | | | |
| *Student* | | | | -0.22 (0.22) | -0.28 (0.22) | -0.21 (0.22) | -0.35 (0.23) | -0.25 (0.22) |
| *Employed* | | | | 0.18 (0.14) | 0.16 (0.14) | 0.18 (0.13) | 0.02 (0.16) | -0.11 (0.15) |
| *Retired* | | | | 0.24 (0.16) | 0.24 (0.16) | 0.25 (0.16) | 0.03 (0.19) | 0.16 (0.18) |
| Family Size (> *3 members)* | | | | 0.07 (0.13) | 0.04 (0.13) | 0.09 (0.13) | 0.03 (0.14) | 0.06 (0.13) |
| Rhône-Alpes | | | 0.13 (0.17) | 0.10 (0.17) | 0.04 (0.13) | 0.12 (0.17) | 0.08 (0.19) | 0.12 (0.18) |
| Awareness of CERN | | | | | 0.23 (0.11)** | | | |
| Scientific interest | | | | | | -0.02 (0.03) | -0.04 (0.03) | -0.03 (0.03) |
| CERN permits an increase in knowledge of universe | | | | | | | 0.41 (0.12)*** | |
| The research activity at CERN should increase in the coming decades | | | | | | | | 0.18 (0.11)* |
| | | | | | | | | |
| Spike | 0.51 (0.01)*** | 0.49 (0.01)*** | 0.49 (0.01)*** | 0.49 (0.01)*** | 0.49 (0.01)*** | 0.49 (0.01)*** | 0.49 (0.02)*** | 0.49 (0.02)*** |
| Observations | 965 | 965 | 965 | 965 | 965 | 965 | 965 | 965 |
| Log- likelihood | -1,069.76 | -1,023.10 | -1,005.28 | -1,002.99 | -1,000.77 | -1,002.84 | -997.44 | -1,001.58 |
| Likelihood ratio test | 250.70*** | 282.47*** | 285.55*** | 285.14*** | 285.60*** | 285.14*** | 286.46*** | 275.71*** |
| AIC | 2143.5 | 2052.2 | 2026.5 | 2030.0 | 2027.5 | 2031.7 | 2022.9 | 2031.2 |
| BIC | 2153.3 | 2066.8 | 2065.5 | 2088.4 | 2090.9 | 2095.0 | 2091.1 | 2099.4 |

*Note.* Standard errors in parenthesis. ***, **, * denote significance at the 1%, 5%, 10% level respectively. Male: omitted category is female; Age (<35): missing category is age > 35; Education:

omitted category is low; Occupation status: omitted category is unemployed; *Family size (>3 member) is*: is family size < 3 members

**Table C**. Correlation between the stated maximum WTP and selected covariates (*N* = 965)

| Variable | Spearman's correlation coefficient |
|---|---|
| Income | 0.19*** |
| Male | 0.10*** |
| Age (< 35) | -0.03 |
| Education | |
| *Low* | -0.06 |
| *Medium* | -0.07 |
| *High* | 0.14*** |
| Occupation status | |
| *Student* | 0.006 |
| *Employed* | 0.04 |
| *Retired* | 0.03 |
| *Unemployed* | -0.09*** |
| Family Size (> *3 members)* | -0.05 |
| Rhône-Alpes | -0.01 |
| Awareness of CERN | 0.20*** |
| Scientific interest | 0.24*** |
| CERN permits an increase in knowledge of universe | 0.22*** |
| The research activity at CERN should increase in the coming decades | 0.30*** |

*Note.* ***, **, * denote significance at the 1%, 5%, 10% level. The Spearman correlation coefficient $(r_s)$ is a statistical measure of the strength of a monotonic relationship between paired data such that $-1 \leq r_s \leq 1$: the closer to $\pm 1$, the stronger the monotonic relationship
