# Supplementary material for: A contingent valuation experiment about future particle accelerators at CERN
Source: PLoS One. 2020 Mar 11;15(3):e0229885. doi: 10.1371/journal.pone.0229885 (PMC7065825; doi:10.1371/journal.pone.0229885)
Supplement: Attachment — Submitted filename: Response to Reviewers.docx [file pone.0229885.s009.docx]

**Please, find in *Italics-blue* our 'Response to Reviewers'. See the file ‘manuscript’ to find the suggested revisions at the corresponding lines.**

Dear Professor FLORIO,

Thank you for submitting your manuscript to PLOS ONE. Let me first warn you that your original editor was unable to complete handling your paper and I stand in. After having read the paper, I had the impression it had an important contribution, even if a bit too deeply buried in the paper structure. This sentiment is shared by the Referees (whose two reports you will find below). Hence, I would like to invite you to revise the paper and resubmit it for PLOS ONE.

In addition to the comments of the Referees, please, revise also the structure of the paper. I admire your drive for transparency and PLOS ONE is definitely not the journal to discourage even lengthy, but important appendices. However, I would advise that the paper starts from the puzzle, states the intended contribution, explains how this contribution is achieved and then discusses the findings. This should be a self-contained unit, from start to end. The rest of your supplementary materials may be relegated to the appendices, but it should be clearly indicated in the main text that a given appendix provides additional insights. At this point, the introduction is too brief (especially for a general audience), and the rest of the paper has an illogical structure (first some results, then some methods, then more results -- confusing. In particular, I am not sure if pre-tests have to be described in such a detail, but I leave it to your discretion, once you revise the main narrative of the paper.

*We revised the structure of the paper as suggested and according to PLOS ONE manuscript organisation instructions: the Introduction, which now better states the research question, followed by Material and Methods, Results and then Discussion and Conclusions. We organised the supporting information in separate files. S1 File contains the description of pilot experiments and pre-tests.*

Also, please, follow PLOS ONE guidelines on paper formatting (referencing in particular)

*Done*

We would appreciate receiving your revised manuscript by Jan 11 2020 11:59PM. When you are ready to submit your revision, log on to <https://www.editorialmanager.com/pone/> and select the 'Submissions Needing Revision' folder to locate your manuscript file.

*The revised manuscript is submitted in compliance with the deadline extension kindly offered by the Editor*

If you would like to make changes to your financial disclosure, please include your updated statement in your cover letter.

*There is no need to modify our financial disclosure*

To enhance the reproducibility of your results, we recommend that if applicable you deposit your laboratory protocols in [protocols.io](http://protocols.io), where a protocol can be assigned its own identifier (DOI) such that it can be cited independently in the future. For instructions see: <http://journals.plos.org/plosone/s/submission-guidelines#loc-laboratory-protocols>

*Anonymized survey data in both Excel and STATA format for the reproducibility of our results will be available at this permanent link:* [*http://www.eiburs.unimi.it/*](http://www.eiburs.unimi.it/)*. All other data and information needed to evaluate the conclusions of this research are included in the paper itself and supporting information.*

Please include the following items when submitting your revised manuscript:

- A rebuttal letter that responds to each point raised by the academic editor and reviewer(s). This letter should be uploaded as separate file and labeled 'Response to Reviewers'.
- A marked-up copy of your manuscript that highlights changes made to the original version. This file should be uploaded as separate file and labeled 'Revised Manuscript with Track Changes'.
- An unmarked version of your revised paper without tracked changes. This file should be uploaded as separate file and labeled 'Manuscript'.

*Done*

Please note while forming your response, if your article is accepted, you may have the opportunity to make the peer review history publicly available. The record will include editor decision letters (with reviews) and your responses to reviewer comments. If eligible, we will contact you to opt in or out.

We look forward to receiving your revised manuscript.

Kind regards,

Joanna Tyrowicz
Academic Editor
PLOS ONE

Journal Requirements:

1. When submitting your revision, we need you to address these additional requirements.
Please ensure that your manuscript meets PLOS ONE's style requirements, including those for file naming. The PLOS ONE style templates can be found at
<http://www.journals.plos.org/plosone/s/file?id=wjVg/PLOSOne_formatting_sample_main_body.pdf> and <http://www.journals.plos.org/plosone/s/file?id=ba62/PLOSOne_formatting_sample_title_authors_affiliations.pdf>

*Done*

2. Please include a copy of the questionnaire in the original language as supporting information, in addition to the English version you already provided.

*Done. In addition to English copy, we Included a copy in French. See S2 File.*

Reviewers' comments:

Reviewer's Responses to Questions

**Comments to the Author**

1. Is the manuscript technically sound, and do the data support the conclusions?

The manuscript must describe a technically sound piece of scientific research with data that supports the conclusions. Experiments must have been conducted rigorously, with appropriate controls, replication, and sample sizes. The conclusions must be drawn appropriately based on the data presented.

Reviewer #1: Yes

Reviewer #2: Yes

2. Has the statistical analysis been performed appropriately and rigorously?

Reviewer #1: Yes

Reviewer #2: Yes

3. Have the authors made all data underlying the findings in their manuscript fully available?

The [PLOS Data policy](http://www.plosone.org/static/policies.action#sharing) requires authors to make all data underlying the findings described in their manuscript fully available without restriction, with rare exception (please refer to the Data Availability Statement in the manuscript PDF file). The data should be provided as part of the manuscript or its supporting information, or deposited to a public repository. For example, in addition to summary statistics, the data points behind means, medians and variance measures should be available. If there are restrictions on publicly sharing data—e.g. participant privacy or use of data from a third party—those must be specified.

Reviewer #1: Yes

Reviewer #2: Yes

4. Is the manuscript presented in an intelligible fashion and written in standard English?

PLOS ONE does not copyedit accepted manuscripts, so the language in submitted articles must be clear, correct, and unambiguous. Any typographical or grammatical errors should be corrected at revision, so please note any specific errors here.

Reviewer #1: Yes

Reviewer #2: Yes

5. Review Comments to the Author

Please use the space provided to explain your answers to the questions above. You may also include additional comments for the author, including concerns about dual publication, research ethics, or publication ethics. (Please upload your review as an attachment if it exceeds 20,000 characters)

Reviewer #1: General comments:
This article provides novel findings on the WTP for basic science in France. It uses an adequate method (CV), carefully administered and pre-tested. The paper is well and clearly written. However, the balance between the main text and the supporting information should be reassessed. In particular, the methodology, descriptive statistics and empirical analysis deserve entire chapters in the main text.

*We appreciate the positive overall assessment of the Reviewer and we are grateful for her/his constructive advice. We have revised the structure of paper as suggested.*

Major comments:
The treatment of protest should be done more carefully, see my comment below.
Methodology and empirical analysis, already present in the supporting information, should be integrated in the main text.
*We have now integrated in the main text the methodology and additional details on the empirical analysis. We accept the suggestion to further discuss the treatment of the (limited number) of “protest” answers, please see below on this specific point.*

Specific comments:
line 115: dropping observations may lead to selection biases. One should be more cautious here. Are protestors and outliers significantly different from "normal" bidders? Do these special characteristics have an impact on WTP? See Garcia et al. (2009) or Borzykowski et al. (2017)

*The issue is discussed in Lines 508-521. We have explicitly mentioned the fact that dropping observations may cause selection bias and cited the references suggested by the Reviewer. Moreover, we now clearly describe the features of protestors and outliers. Protestors are indeed different from "normal" bidders because of their overall negative beliefs about any international organizations and government-funded programs. This negatively impacts on the mean WTP, but clearly these responses are unrelated to the specific experiment, as their reasons would have a negative impact on any project supported by public funds [see for instance reference 35 in the text]. In contrast, outliers seem to be very generous people, i.e. those people with a ratio between the stated maximum WTP and their income higher than 0.5%. While these responses may reflect true preferences, we excluded them from the WTP calculation because we want to be conservative.*

*In any case, we tested the impact of our (modest) sample trimming on the mean WTP by performing supplementary analyses reported in S6 File, S5 Table. We show that the effect of these adjustments, which are in compliance with best practice in the field, is however negligible.*

lines 124 to 128: This formulation is too close from Borzykowski et al. (2018), please modify or quote.
*Thank you for suggesting a different wording. See lines 531-535. We have now re-formulated the sentence.*

Line 158: Why do you talk about scope test here? Did you perform any? Do you analyse internal consistency later? I know that you do, but it is not clearly stated in the text, so please explain exactly what you consider to be an internal consistency test and later in the text, please specify if you find consistency or not.
*See lines 552-570. We have now clearly stated what a scope test is in our context. We have also explained that exploring the impact of socioeconomic traits, interest, knowledge, and attitudes towards CERN and its research activity on the mean WTP is a way to show internal consistency of our experiment. Later in the text, after the statistical analysis, we specified that our results are consistent with economic and social psychological research (line 673). In fact, our paper goes much beyond the usual testing for internal consistency in earlier literature, because we wanted to be sure that the stated preferences are coherent with a set of individual characteristics, such as attitudes about science and education, as in fact they are.*

line 165: What do you mean by "tested bivariate relationships"? Did you perform a linear regression between 2 variables or just correlation? Please explain
*Thank you for asking this question. See lines 667-670. We performed correlation by resorting to the Spearman’s rho correlation coefficient. We have now specified it in the text.*

line 167: Why is the relationship between income and WTP for a public good unexpected? Please explain
*This is an interesting issue that we have now clarified. See lines 624, 684-692, 712. In the text, now we just say that the WTP is positively and significantly correlated with income. We specify, however, that as we consider a pure public good, whether or not considering income elasticities to infer the WTP remain an issue since for public goods we do not necessarily expect WTP to have as large as income elasticity as we would with respect to demand for a private good [see for instance reference 43 for a discussion]. See also the further discussion below.*

line 178: Is awareness of CERN linked with the place respondents live? I assume that, the closer they live, the more aware they. This would lead to an issue of colinearity, which could explain the non-significant impact of distance. Don't you have a way to be more specific regarding the place of residence? In particular for face-to-face interviews, I assume that you have addresses.
*See lines 719-720. Awareness of CERN and the place the respondents live are not correlated. For instance (this not reported in the text) we computed the odds ratio between the variables “Awareness of CERN” and “Rhône-Alpes”, which is the region that hosts CERN. The odds ratio is 1.01 with a p-value equals to 0.94, suggesting absence of statistical association between these two variables. This is corroborated by the Spearman’s rho (=0.0022; p-value=0.94). As matter of fact, in the region of Rhône-Alpes, almost half sample (47% of respondents) was aware of CERN; in contrast, the other half (53%) was not aware of what CERN is.*

*As regards the place of residence of respondents, we (as University of Milan) do not have access to individual respondents addresses because of privacy reasons. Eumetra (the polling company) provided us with pseudonymised data in compliance with CERN’s Operational Circular n° 11 (OC11) on the processing of Personal Data (*[*https://home.cern/news/official-news/cern/operational-circular-no-11-processing-personal-data-cern*](https://home.cern/news/official-news/cern/operational-circular-no-11-processing-personal-data-cern) *) (line 226) and with the EU General Data Protection Regulation (GDPR). We have however the region of residence, which has allowed us to perform the above mentioned geographical analysis.*

line 192: I am surprised by the fact that the DBDC gives much smaller WTP. Any hypothesis why? What distributional assumption would you consider better?
*Thanks for asking this question. As far as we know, the fact that the DBDC gives smaller WTP than the Spike model is not uncommon in CV studies (see for instance [25, Table 1]). When there is a high share of zero responses in the sample, conventional models using symmetric distributional assumptions such as the DBDC or the SBDC produce biased positive small or negative estimations of the mean WTP (see for instance [33]). This because such models cannot by design properly fit the distribution of data. When the WTP distribution is asymmetric, mixture models, such as the spike one is recommended. The spike model takes into account the spike at zero. i.e. the truncation, at zero, of the negative part of the WTP distribution. Accordingly, we consider better the spike distributional assumptions, and this was also suggested to us by some of the experts in the field we have asked to comment on previous versions (their names are now acknowledged in this version). We discussed all this in the lines 375-382; 527-535.*

line 249 and following: Was you sample representative of the student population?
*See S1 File (line 14). Our sample in the pilot experiments was not representative of the student population. The objective was not inference but testing the understand of the questions and other technical aspect of the CV experiment. We specified this in the S1 File.*

line 273: Why and how were respondent likely to react differently with lump-sum vs. annual payments?
*This is an interesting question, that we now discuss in the revised paper. See S1 File (lines 36-42). Several CV papers have investigated the reactions of respondents to different payment systems. They show that it is a matter of perceptions, individual time preferences and implicit discount rates [45, 46, 47]. The authors of certain experiments with students argued that this difference could be attributable to the fact that many students have weekly budget constraints; so, weekly payments are likely to be more reliable than long-term payment schedules, with which students often have relatively little experience.*

*In our specific case, out of 1,022 undergraduate students, only 30% of them earned their own income. So,* *although several factors may be responsible, in our opinion the preference for smaller annual payments with respect to a larger lump-sum payment was just the result of a mental accounting [se for instance 47].* *Budget constraints are likely to constrain in either case; however, respondents feel less bound when they are asked smaller ongoing payments rather than larger lump-sum disbursement. An additional reason may be that, when dealing with donations to charities or organisations, included science-related ones, people are used to donating a fixed amount each year instead of a large one-time payment.*

line 283: "Would you BE willing to pay EUR" the "be" is missing
*Done, see S1 File (line 52).*

line 301: What do you mean by "binomial logit model with numerical integration"? What is the numerical integration about?

*See S1 File (lines 70-71). We removed the above sentence and only stated “logit model”. The numerical integration is explained in line 73.*

line 479: How do you get a 100% response rate? This is misleading or doubtful
*See line 430. We removed the sentence from the text.*

line 483-486: Not clear. Please rephrase
*These lines were meant to discuss the transfer of the results to other contexts or CERN Member States. With the new structure of the paper, this issue in discussed in the conclusions. So, we removed these lines from the text.*

line 603: Please cite Borzykowski et al. (2018) here.
*Done. See line 371.*

line 650: This estimator is affected by the anchor of the bids. How do you treat this issue? If you don't, you need to.
*See lines 521-523. We made explicit that S6 File, S6 Table contains additional analysis, i.e. the estimation of the mean WTP by using the SBDC-CV procedure, which is less exposed to anchoring.*

line 805: What robustness test are you talking about? Statistically, you cannot reject the alternative hypothesis. You do not reject the null hypothesis of mean equality.
*Now S6 File (lines 31-33). We specified that we referred to both Pearson's chi-square tests (when considering the spike-model) and t-tests (for the maximum WTP) and, as suggested, we wrote that we “do not reject the null hypothesis of mean equality”.*

Tables S6, s7: What is the preferred model? Could you provide the AIC / BIC and discuss it in the text?

*See Table 3 line 645; and Table 4, line 662. We provided both AIC and BIC and their interpretation (lines 642-644).*

*However, as we explained in lines 684-686, while extended models with covariates are useful tools to test the determinants of WTP, simple specifications including only the bid and income remain our preferred ones to infer the mean WTP for research at CERN. Such specifications are the most used in the CV literature to elicit the WTP because essentially validate the basic axioms of preferences, including the non-increasing relationship between the proportion of people who would to pay and the amount of bid asked and the relevance of budget constraints [16]. Actually, as we have a pure public good, whether considering income elasticities to infer the WTP is tricky since for public goods we do not necessarily expect WTP to have as large as income elasticity as we would with respect to demand for a private good [43]. For this reason, throughout the paper we mainly estimated the WTP from specifications including only the bid variable.*

Table S7: How do you explain the positive impact of the bid variable? What does this mean and how do you get positive WTP afterwards?
*Now Table 4, line 662. We explained this in the Paragraph “the Spike model”, Eq. 12.*

Table s8: How were the CI computed?
*See S6File, S5 Table. We used the “bootstrap” command in STATA, with the respective sample size as reported in Column 2 and using 1,000 replications.*

References:
Borzykowski, N., Baranzini, A., & Maradan, D. (2017). Y a-t-il assez de réserves forestières en Suisse? Une évaluation contingente. Économie rurale. Agricultures, alimentations, territoires, (359), 51-79.
Borzykowski, N., Baranzini, A., & Maradan, D. (2018). Scope effects in contingent valuation: does the assumed statistical distribution of WTP matter?. Ecological Economics, 144, 319-329.
Garcia S., Harou P., Montagné C., Stenger A. (2009). Models for sample selection bias in contingent valuation: Application to forest biodiversity. Journal of Forest Economics, vol. 15, n° 1, pp. 59-78.
DOI : 10.1016/j.jfe.2008.03.008
Meyerhoff J., Mørkbak M.R., Olsen S.B. (2014). A meta-study investigating the sources of protest behaviour in stated preference surveys. Environmental and Resource Economics, vol. 58, n° 1, pp. 35-57.
DOI : 10.1007/s10640-013-9688-1
Strazzera E., Genius M., Scarpa R., Hutchinson G. (2003). The effect of protest votes on the estimates of WTP for use values of recreational sites. Environmental and Resource Economics, vol. 25, n° 4, pp. 461-476.

Reviewer #2: The article is very interesting and it offers a novel approach to study attitudes to science and technology. It also includes a thorough description of the design, methodology and implementation procedure.

*We appreciate this overall positive and encouraging comment.*

Nevertheless, the statistical models are not clearly enough defined. It is possible that they are evident for readers familiarized with stated prefernces techniques, Contingent Valuation and the utility-theoretic framework, but not for researchers not familiarized with them. The intelligibility and interest of the article will improve with a brief and clear description of the models and of the utility-theoretic framework.

*We are grateful for this advice and, as suggested, we integrated in the main text a detailed and clearer description of the CV technique and the utility-theoretic framework underlying the econometric analysis.*

On the other hand, in Table S5, the authors provide the descriptive statistics of the variables of what they call the "Econometric analysis". It is quite strange and disinformative read that the variable "male" has a mean of 0.49 with a Standard Deviation of 0.50, that the mean of high education is 0.33 with a Standard Deviation of 0.47, etc. This may be a well stablished procedure in econometric analysis, but descriptive statistic clearly stablish that the mean and standard deviation, as measures of central tendency, are only appropriate to describe quantitative variables (continuous and discrete) but not qualitative ones (dichtomous and ordinals). Although not the best election, the mean and standard deviation only may be considered informative in describing scientific interest, as this variable is ordinal with five levels. Obviously, this comment is "anechdotic" and, hence, does not discredit the high quality of the article, but it is advisable that the descriptive statistic adjusts to the measuring level of the variable.

*We removed the column with the Standard Deviation from the Table. We integrated the new table in the main text as Table 2 line 615, which reports the Mean, Min and the Max.*

Finally, another to some extent irrelevant for the final results of the article, but somewhat disconcerting issue, is the decision to measure attitudes with a five points scale with a midpoint defined as neutral, to finally collapse the five categories into two, combining the neutral point with the "negative" categories (disagree and strongly disagree). First, if it is properly a neutral category, it should be considered equidistant to the positive and negative poles of the scale and thus might be logically included in the positive section of the scale. Second, there are founded doubts in the literature concerning the real meaning of the neutral midpoint in attitude questions, as it may represents a combination of actual neutral responses, covert "I don't know" answers, or a satisficing strategy: the tendency to provide a reasonable response, not the best one; that is to say, the respondents tendency to select the "easy" option.

*Thanks for this comment. The usage of a point scale is standard in CV literature, but we agree that the interpretation of neutral responses is always tricky, in any context. We modified all the figures to show the percentages of the neutral responses. See S5 File, Figs S2, S3, S4.*

6. PLOS authors have the option to publish the peer review history of their article ([what does this mean?](https://journals.plos.org/plosone/s/editorial-and-peer-review-process#loc-peer-review-history)). If published, this will include your full peer review and any attached files.


If you choose “no”, your identity will remain anonymous but your review may still be made public.


**Do you want your identity to be public for this peer review?** For information about this choice, including consent withdrawal, please see our [Privacy Policy](https://www.plos.org/privacy-policy).

Reviewer #1: No

Reviewer #2: No


[NOTE: If reviewer comments were submitted as an attachment file, they will be attached to this email and accessible via the submission site. Please log into your account, locate the manuscript record, and check for the action link "View Attachments". If this link does not appear, there are no attachment files to be viewed.]


While revising your submission, please upload your figure files to the Preflight Analysis and Conversion Engine (PACE) digital diagnostic tool, <https://pacev2.apexcovantage.com/>. PACE helps ensure that figures meet PLOS requirements. To use PACE, you must first register as a user. Registration is free. Then, login and navigate to the UPLOAD tab, where you will find detailed instructions on how to use the tool. If you encounter any issues or have any questions when using PACE, please email us at [figures@plos.org](mailto:figures@plos.org). Please note that Supporting Information files do not need this step.
