# Supplementary material for: A contingent valuation experiment about future particle accelerators at CERN
Source: PLoS One. 2020 Mar 11;15(3):e0229885. doi: 10.1371/journal.pone.0229885 (PMC7065825; doi:10.1371/journal.pone.0229885)
Supplement: Attachment — Submitted filename: Response to Reviewers.docx [file pone.0229885.s010.docx]

**Please, find in *Italics-blue* our 'Response to Reviewers'. See the file ‘manuscript’ to find the suggested revisions at the corresponding lines.**

PONE-D-19-28867R1
A contingent valuation experiment about future particle accelerators at CERN
PLOS ONE

Dear Professor FLORIO,

thank you for submitting your revision to PLOS ONE. In the interest of clarity and readability of your text, I suggest two following changes.

Change #1. In the final part of your empirical analysis, you report three separate tables, each with full set of specifications, to make only a few notes. I find this ok (no need to make more points!), but perhaps it would be easier for the readers to follow your line of reasoning if you report a plot of marginal effects from all three outcome measures in one graph, space in the graph split by variables of interest. I have in mind something like Figure 7 in this source (if you use STATA: <https://www.stata.com/meeting/germany14/abstracts/materials/de14_jann.pdf>) or predicting gas mileage plot (if you use R: <https://cran.r-project.org/web/packages/dotwhisker/vignettes/dotwhisker-vignette.html>). It can be vertical or horizontal, but my point is to shorten three tables to one graph that has all the content you require. You can (and should) relegate the full tables to the online appendix then.

***We thank you very much for this suggestion. We summarised the result of our empirical analysis in Fig. 6 as you suggested. However, the layout and the readability of the Fig 6 resulted very bad when all the three models were jointly plotted in, so we decided to only visualise in the main text results from our two most important models: the double bounded dichotomous choice (DCDB-CV) and the Spike models. As you indicated, we relegated the full set of estimates, including the ones from our third model (i.e. the MAX WTP), in the on-line appendix “S7 File. Drivers of WTP”.***

Change #2. You use frequently the abbreviation DBDC-CV without ever (I think ever?) explaining it to the reader. Please, make the courtesy to the reader to make it clear below every table or figure what each abbreviation signifies, so that one did not have to browse through the whole text to find the meaning.

***We did it throughout the paper. Moreover, to maintain the layout and readability of the new Fig.6, we explicated the abbreviation DBDC-CV in the description of Fig. 6. See line 644-647.***

Overall, I cannot ask you to make those changes other than through minor revision option, I hope you do understand that the changes I propose above are in the interest of making your paper more reader-friendly. You may consider your paper accepted, conditional on those minor changes.

We would appreciate receiving your revised manuscript by Mar 27 2020 11:59PM. When you are ready to submit your revision, log on to <https://www.editorialmanager.com/pone/> and select the 'Submissions Needing Revision' folder to locate your manuscript file.

If you would like to make changes to your financial disclosure, please include your updated statement in your cover letter.

To enhance the reproducibility of your results, we recommend that if applicable you deposit your laboratory protocols in [protocols.io](http://protocols.io), where a protocol can be assigned its own identifier (DOI) such that it can be cited independently in the future. For instructions see: <http://journals.plos.org/plosone/s/submission-guidelines#loc-laboratory-protocols>

Please include the following items when submitting your revised manuscript:

- A rebuttal letter that responds to each point raised by the academic editor and reviewer(s). This letter should be uploaded as separate file and labeled 'Response to Reviewers'.
- A marked-up copy of your manuscript that highlights changes made to the original version. This file should be uploaded as separate file and labeled 'Revised Manuscript with Track Changes'.
- An unmarked version of your revised paper without tracked changes. This file should be uploaded as separate file and labeled 'Manuscript'.

Please note while forming your response, that you may have the opportunity to make the peer review history publicly available. The record will include editor decision letters (with reviews) and your responses to reviewer comments. If eligible, we will contact you to opt in or out.

We look forward to receiving your revised manuscript.

Kind regards,

Joanna Tyrowicz
Academic Editor
PLOS ONE
